# Effect of the Positioning of Metal Centers on a Cavitand in the Ruthenium-Catalyzed *N*-Alkylation of Amines

**DOI:** 10.3390/molecules30040951

**Published:** 2025-02-18

**Authors:** Neslihan Şahin, Christophe Gourlaouen, David Sémeril

**Affiliations:** 1 Department of Science Education, Faculty of Education, Cumhuriyet University, Sivas 58040, Türkiye; 2 Synthèse Organométallique et Catalyse, UMR-CNRS 7177, Strasbourg University, 67008 Strasbourg, France; 3 Laboratoire de Modélisation et Simulations Moléculaires, UMR-CNRS 7140, Strasbourg University, 67008 Strasbourg, France

**Keywords:** ruthenium, resorcin[4]arene, cavitand, hydrogen borrowing, cooperative effect, mechanistic computation

## Abstract

Two bis-ruthenium(II) complexes, namely *N*,*N′*-{5,17-diamino-4(24),6(10),12(16),18(22)-tetramethylenedioxy-2,8,14,20-tetrapentylresorcin[4]arene}-bis-[dichloro-(*p*-cymene)-ruthenium(II)] (**1**) and *N*,*N′*-{5,11-diamino-4(24),6(10),12(16), 18(22)-tetramethylenedioxy-2,8,14,20-tetrapentylresorcin[4]arene}-bis-[dichloro-(*p*-cymene)-ruthenium(II)] (**2**) were synthesized and tested as catalysts in the *N*-alkylation of primary amines with arylmethyl alcohol using the green “hydrogen borrowing” methodology. The catalytic results were compared with those obtained when the *N*-{5-amino-4(24),6(10),12(16),18(22)-tetramethylenedioxy-2,8,14,20-tetrapentyl-resorcin[4]arene}-[dichloro-(*p*-cymene)-ruthenium(II)] (**3**) complex was employed as catalyst. The rate of the *N*-alkylation of aniline with benzyl alcohol increased in the order **3** < **1** ≪ **2**, which highlights the importance of the relative positioning of the two metal centers on the upper rim of the resorcin[4]arene. Theoretical investigations suggest that the grafting of the two “RuCl_2_(*p*-cymene)NH_2_” moieties on two distal aromatic rings of the cavitand allows a cooperative effect between a ruthenium atom and the coordinated amine of the second metal center.

## 1. Introduction

Resorcinarenes, first studied by Baeyer in the late 19th century [1], are a class of macrocycles obtained by the condensation of resorcinol and aldehyde in EtOH/H_2_O under acidic conditions. The thermodynamic product, which is usually obtained in high yields, comprises four units of resorcinol and aldehyde [2]. These macrocycles are flexible and exist in various conformations in equilibrium. However, they can be easily rigidified by linking neighboring hydroxyl groups with short methylene bridges, thus forming what Cram called cavitands [3,4].

**Figure 1:**
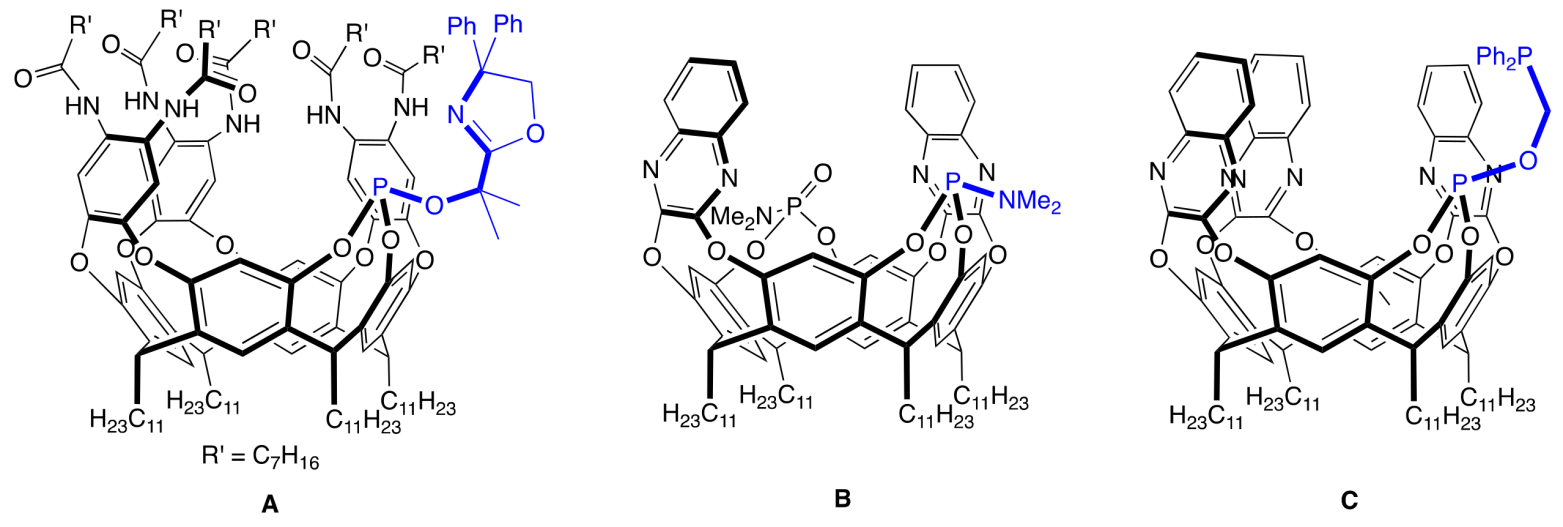
Selected examples of cavitands (**A**–**C**) able to influence the catalytic outcome.

Cavitands can be easily functionalized [5,6], and a large variety of coordinating functions can be introduced, in particular phosphorus or nitrogen derivatives and *N*-heterocyclic carbenes [7,8]. This preorganization platform has led to sophisticated catalysts [9,10,11,12,13] as demonstrated by Gibson and Rebek with their expanded cavitand **A**, which is able to form *P*,*N*-chelate (Figure 1) [14]. In the palladium-catalyzed allylic alkylation with dimethylmalonate, the partial entrapment of the substrate in the pocket formed by the cavitand favors nucleophilic attack on the less sterically carbon atom, exclusively leading to the formation of the linear products. Iwasawa and co-workers observed that in the gold-catalyzed hydration of 1-butynylbenzene with the cavitand **B**, which has phosphoramidite and phosphoramidate moieties, 98% of 1-phenyl-2-butanone was formed, with only 2% of 1-phenyl-1-butanone (Figure 1) [15]. Notably, in the test with cavitand devoid of phosphoramidate substituent, no conversion of the alkyne was observed. The authors confidently interpreted these results as an activation of the water molecule by the ideally positioned P=O group, which facilitates its addition to the alkyl bond. Chaplin and co-workers recently reported a tris-quinoxaline extended resorcin[4]arene on which a bis-phosphinated substituent (phosphite-phosphine) was grafted (**C**; Figure 1) for the rhodium-catalyzed hydroformylation of terminal alkenes [16]. The hydroformylation of octene under 20 bar of syngas at 60 °C with the [Rh(acac)**C**] (acac = acetylacetonate) complex resulted in a 5.9 times higher proportion of branched aldehyde than linear. The unusual regioselectivity was explained by the authors by the steric constraints imposed by the quinoxaline walls on the catalytic center. These constraints, during the insertion step of the olefin in the Rh-H bond, result in the formation of secondary alkyl intermediate, rather than the linear one.

**Figure 2:**
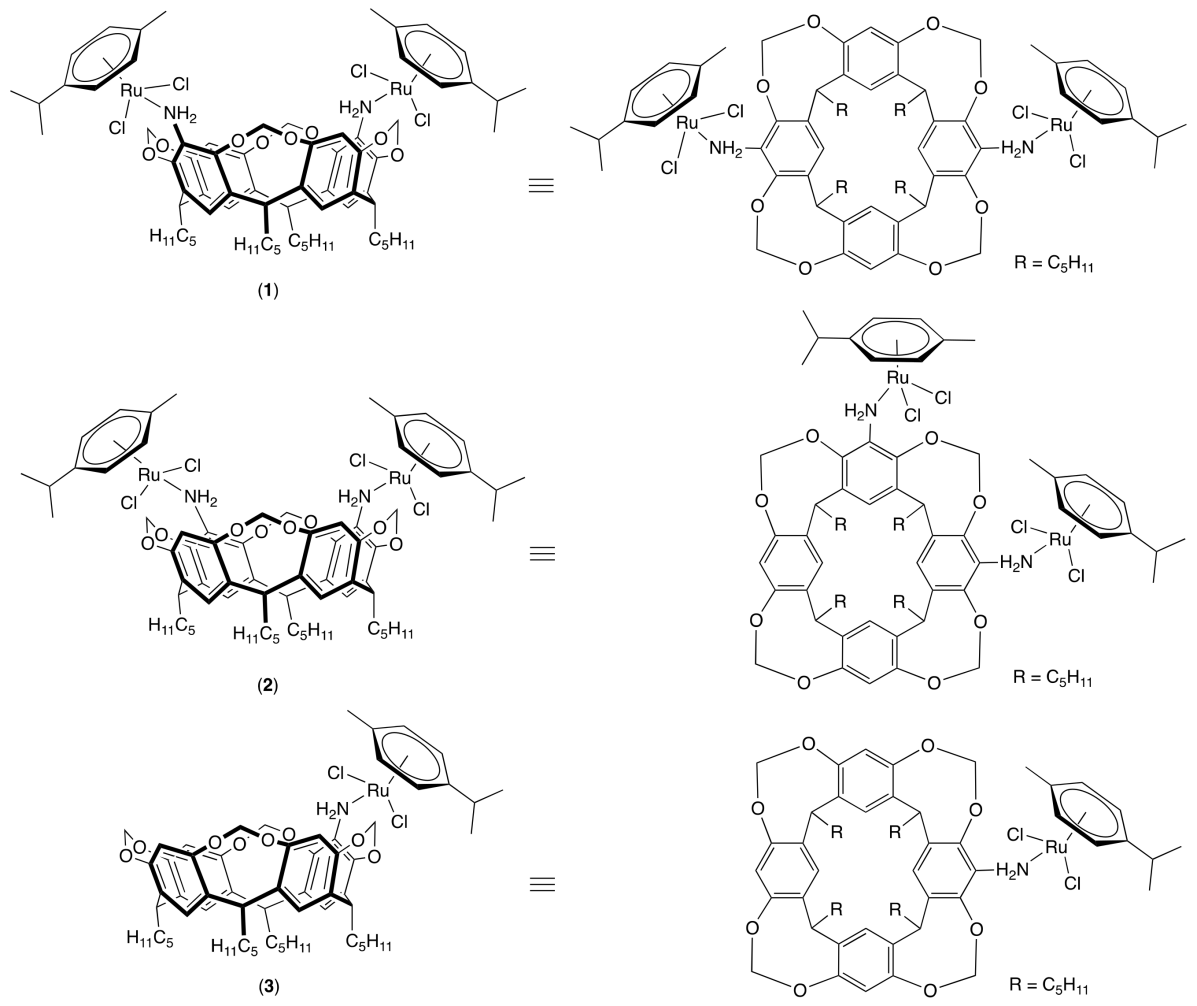
Ruthenium complexes **1**–**3** used in the present study.

In this context, we report on the synthesis of ruthenium complexes anchored on the upper rim of the cavitand, in which the metallic atoms are grafted on two distal (**1**) and proximal (**2**) aromatic rings. This results in complexes in which the two metal centers are close (ruthenium atoms on proximal aromatic rings—**2**) or distant (ruthenium atoms on distal aromatic rings—**1**). The catalytic results of these bis-ruthenium systems are ranked with the analogous mono-ruthenium complex **3** (Figure 2). As we have seen before with phosphinated calix[4]arenes [17] or resorcin[4]arene [18], the position of the phosphorus atoms on the macrocyclic platform has a significant impact on the coordination sphere of the metal and the resulting catalytic outcome. The aim of this study is to highlight the consequences of a structural modification in the active species, the change in the positioning of the metal centers on the macrocyclic platform and on the *N*-alkylation of primary amines with a primary alcohol through synergy between nearby catalytic centers. The C-N bond will be formed using the green “hydrogen borrowing” methodology. This consists, firstly, of alcohol dehydrogenation, which results in the corresponding aldehyde. In this step, two hydrogen atoms of the reactant are temporarily transferred to the active species. In the second step, the formed aldehyde condenses with the primary amine to generate the intermediate imine. Finally, the active species is regenerated by the hydrogenation of the C=N bond (transfer of the two hydrogen atoms from the catalytic intermediate to the organic substrate) to form the *N*-alkylated amine. This reaction can be carried out with cobalt, iridium, iron, manganese, palladium, platinum, rhodium, etc., catalysts; however, in the present study, we focus on ruthenium-based catalysts [19,20,21,22].

## 2. Results and Discussion

### 2.1. Synthesis of the Cavitands and Their Ruthenium Complexes

**Scheme 1:**
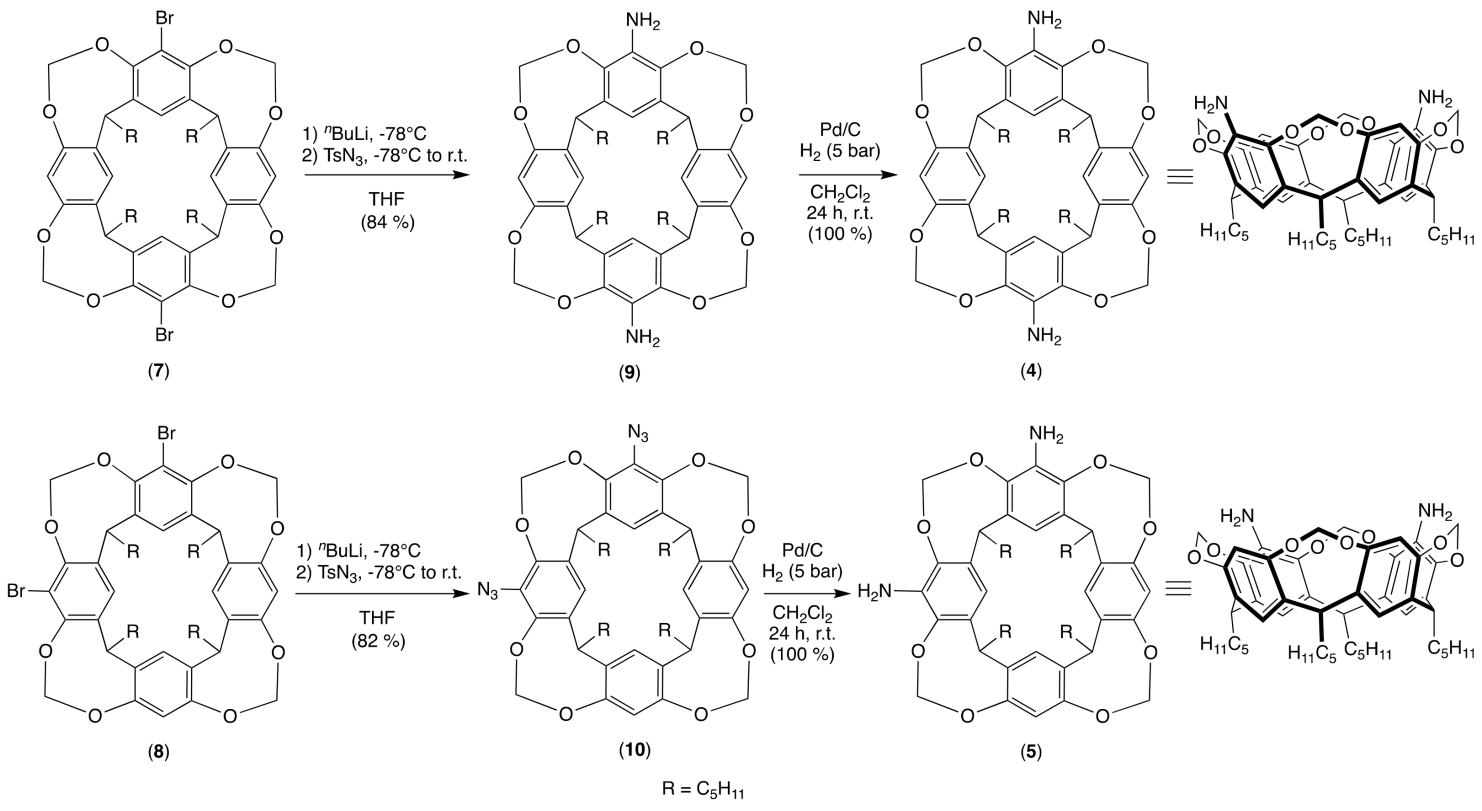
Synthesis of resorcinarenyl bis-amines **4** and **5**.

In the first step, we prepared the two cavitands, namely 5,17-diamino-4(24),6(10),12(16),18(22)-tetramethylenedioxy-2,8,14,20-tetrapentyl-resorcin[4]arene (**4**) and 5,11-diamino-4(24),6(10),12(16),18(22)-tetramethylenedioxy-2,8,14,20-tetrapentyl-resorcin[4]arene (**5**), substituted by two amine functions, in distal and proximal positions, respectively, in two steps from the corresponding bis-brominated resorcinarenes **7** and **8** (Scheme 1).

The two cavitands were conveniently prepared according to the method reported earlier for 5-amino-4(24),6(10),12(16),18(22)-tetramethylenedioxy-2,8,14,20-tetrapentyl-resorcin[4]arene (**6**) [23]. The bis-brominated cavitands were converted into azide derivatives **9** and **10**, in 84 and 82% yield, respectively. This was achieved through a halogen/lithium exchange followed by reaction with tosyl azide, which were then quantitatively reduced with Pd/C under 5 bar of hydrogen in bis-amines **4** and **5**. Cavitands **4** and **5** have the same crude formula, but their ^1^H NMR spectra clearly differentiate them. The *C*_2v_-symmetry of **4** indicates the presence of a unique AB patterns at 5.79/4.42 ppm for the four OCH_2_O groups. In contrast, the *C*_s_-symmetry of **5** is characterized by three AB patterns at 5.84/4.32, 5.79/4.41 and 5.74/4.51 ppm (intensity 1:2:1).

**Scheme 2:**
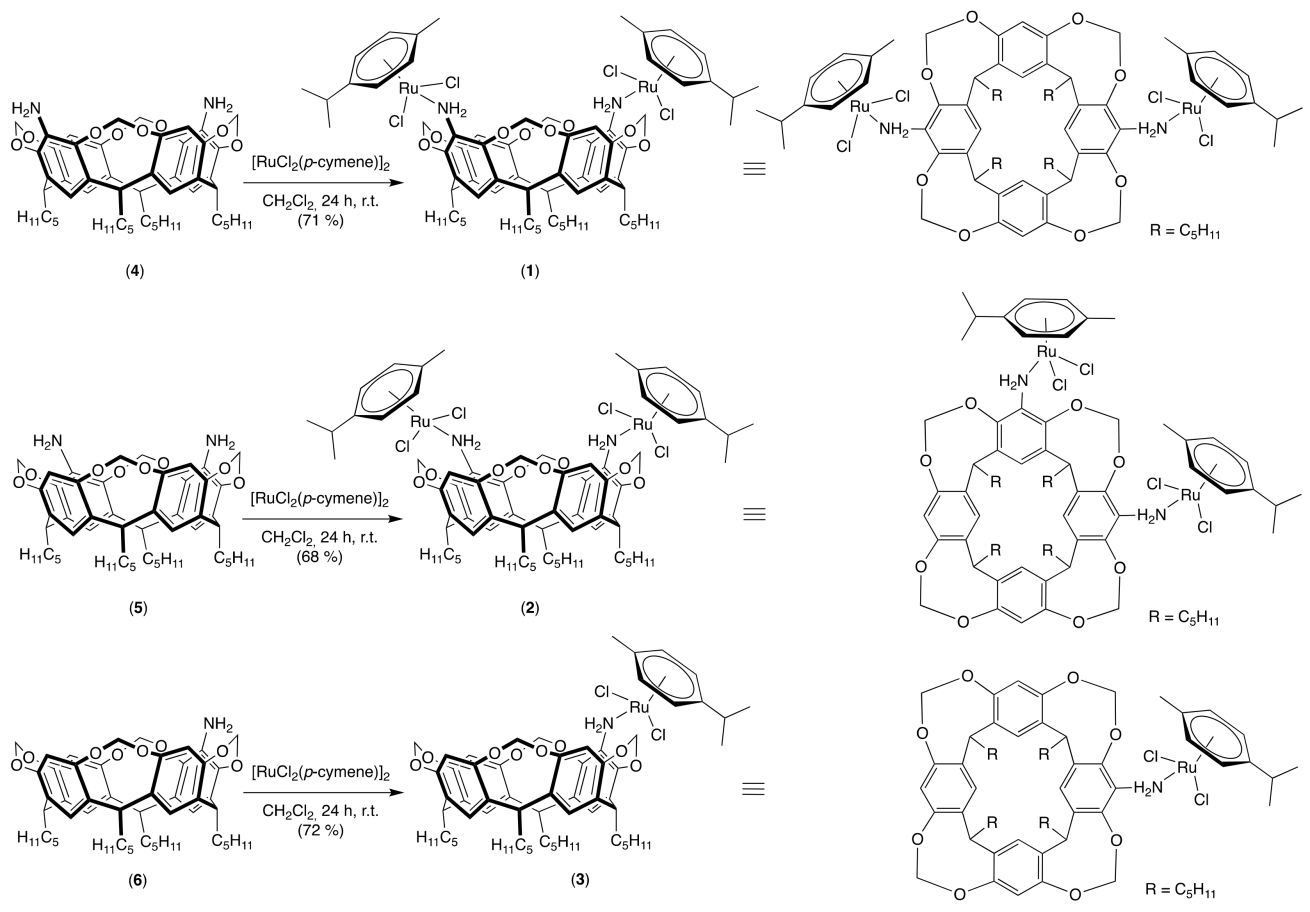
Synthesis of ruthenium complexes **1**–**3**.

In the second step, the dimeric [RuCl_2_(*p*-cymene)]_2_ precursor reacts with anilines to form the corresponding [RuCl_2_(*p*-cymene)(H_2_NAr)] complexes, in which the ruthenium is coordinated to the nitrogen atom [24,25,26,27]. The ruthenium complexes **1–3** were obtained at a 68–72% yield by the reaction of the amino-cavitand and the [RuCl_2_(*p*-cymene)]_2_ precursor (Scheme 2). They were fully characterized using elemental analysis, NMR, and mass spectroscopy. The symmetry of the complexes is identical to that of the corresponding free ligands **4**–**6**. Complex **3**, with a single metal center, unquestionably displays two AB systems at 5.58/4.38 and 5.58/4.31 ppm for the four OCH_2_O groups. The ^1^H NMR spectra of the bis-metal complexes **1** and **2** are less well resolved than that of complex **3**, which suggests that the presence of two “RuCl_2_(*p*-cymene)” moieties severely restrict free rotation. The mass spectra analysis of the three complexes confirmed the formation of the targeted complexes. Each organometallic compound has a peak, *m*/*z* = 1423.42 for **1** and **2** and 1102.45 for **3**, corresponding to [M − Cl]^+^ cations with the expected isotopic profiles.

### 2.2. X-Ray Crystal Structure Analysis

**Figure 3:**
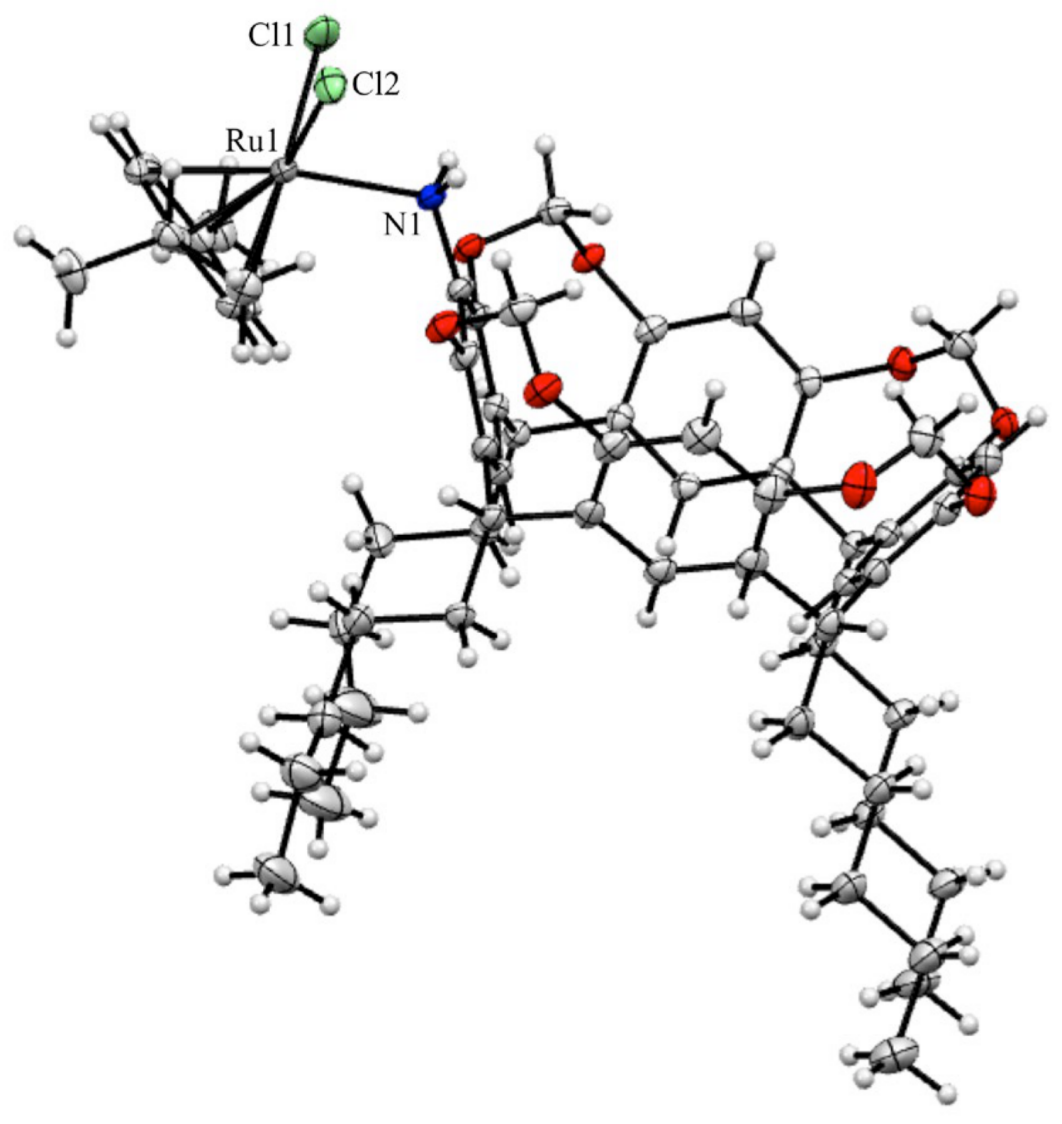
ORTEP drawing of ruthenium(II) complex **3**, with a 50% probability thermal ellipsoids. For clarity, the 1.15 molecules of chloroform are not represented. Important bond lengths (°) and angles (◦): N1-Ru1 2.1719(18), Ru1-Cl1 2.3958(6), Ru1-Cl2 2.4001(6), N1-Ru1-Cl1 82.93(6), Cl1-Ru1-Cl2 86.75(3), and Cl2-Ru1-N1 83.28(5).

Single crystals of ruthenium(II) complexes **1** (see Supplementary Materials) and **3** (Figure 3), which were suitable for X-ray analysis, unambiguously confirming the formation of the *N*-coordinated metal complexes. This complex **1** crystallizes in the triclinic space group *P*-1. The “RuCl_2_(*p*-cymene)” moiety is *exo*-oriented toward macrocyclic cavity, which hosts a molecule of CHCl_3_. The *p*-cymene ligand (C53-C62) is inclined relative to the aromatic ring of the cavitand with a dihedral angle of 24.58°. The aromatic ligand is disordered over two positions with a ratio of 0.85/0.15. Chloroform is positioned nearby in the minority position, contributing 0.15 molecules of chloroform to the structure. The ruthenium atom adopts a typical pseudo-octahedral geometry with the *p*-cymene occupying three adjacent sites of the octahedron with a Ru-centroid *p*-cymene length of 1.670 Å. The remaining three sites are occupied with two chloride atoms (bond lengths of Ru-Cl = 2.3958(6) and 2.4001(6) Å) and the amine of the cavitand (bond length of Ru-N = 2.1719(18) Å). These lengths are in line with those previously reported for [RuCl_2_(*p*-cymene)(H_2_NAr)] complexes, as in *N*-aniline-[dichloro-(*p*-cymene)-rutheni-um(II)] [24] or in *N*-(4-trifluoromethoxy)aniline-[dichloro-(*p*-cymene)-ruthenium(II)] [27] complexes.

### 2.3. Catalytic Tests

**Scheme 3:**
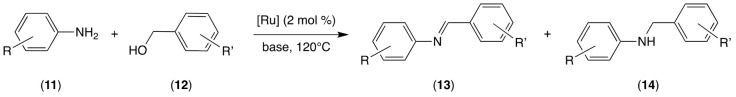
Ruthenium-catalyzed *N*-alkylation of amines.

The *N*-coordinated ruthenium complexes **1**–**3** were tested in the *N*-alkylation of amines (**11**) with alcohols (**12**) using the “hydrogen borrowing” method. In this catalytic reaction, in addition to the expected amine derivatives (**14**), intermediate imines (**13**) can be observed (Scheme 3).

To determine optimum reaction conditions, tests were carried out with aniline and benzyl alcohol as a model reaction using 2 mol% of complex **3** for 3 h at 120 °C. Using *^t^*BuOK as base, three classical solvents [27] were tested, namely toluene, DMF (*N*,*N*-dimethylformamide), and 1,4-dioxane. The chromatogram analysis of the latter reaction mixtures clearly showed that a high proportion of imine (ratio amine/imine up to 6/94) was formed, especially when toluene was employed. The large formation of *N*,1-diphenylmethanimine implies that the last step in the catalytic cycle, the hydrogenation of the imine, did not occur (Table 1, entries 1–3). Repeating the run in solvent-free conditions drastically changed the chemoselectivity of the reaction. After 3 h at 120 °C, *N*-benzylaniline was formed with an amine/imine ratio of 92/8 (conversion of aniline of 40%; Table 1, entry 4). To maximize the formation of *N*-benzylaniline, the following reactions were carried out without solvent.

In a second series of runs, four widely utilized bases [27,28] (KOH, K_2_CO_3_, Cs_2_CO_3_, and NaOAc) were compared to *^t^*BuOK (Table 1, entries 5–9). After a heating period of 6 h, the results showed that the highest conversion of aniline was observed when K_2_CO_3_ was employed (75%; Table 1, entry 6). Unfortunately, under these conditions, the chemoselectivity was strongly in favor of the imine. The best compromise in terms of efficiency and chemoselectivity was achieved when *^t^*BuOK was used (60% conversion and amine/imine ratio of 93/7; Table 1, entry 9).

The three ruthenium complexes **1**–**3** were finally compared in the following tests, with a catalytic loading of 2 mol% of ruthenium atom (1 mol% of complexes **1** and **2**; 2 mol% of complex **3**). The three runs yielded a significant amount of *N*-benzylaniline, with amine/imine ratios ranging from 80/10 to 98/2. The conversions increased in the following order **3** < **1** << **2**; this proved that the pre-catalysts with two metal centers (**1** and **2**) are more efficient than their mono-metallic homologue **3**. We also observed that the positioning of the two metals on the macrocyclic platform is significant. The data clearly show that when the two catalytic centers are grafted onto proximal aromatic rings (complex **2**), conversions are higher (82%) than in catalytic centers grafted onto distal aromatic rings (complex **1** and 65% of conversion). The conversion with the latter bis-ruthenium system is only slightly higher that observed in the mono-metallic complex **3** (Table 1, entries 9–11).

By comparison, using a pre-catalyst similar to our complex **2**, the *N*-(4-trifluoromethoxy)aniline-[dichloro-(*p*-cymene)-ruthenium(II)] complex (**D**), Vyas and co-workers observed after 6 h at 110 °C only the formation of *N*-benzylaniline (conversion of 78%); however, a significant proportion of benzaldehyde (22%) was also formed [27] (Table 1, entry 12). We demonstrated that the use of *N*-heterocyclic carbene ligands, the dichloro-[1-(3,3-dimethylallyl)benzimidazole]-(*p*-cymene)-ruthenium(II) (**E**) [28] and dichloro-{1-[2-(2-ethoxyphenoxy)ethyl]-3-(3,5-dimethylbenzyl)benzimidazol-2-ylidene}(*p*-cymene)-ruthenium(II) (**F**) [29] complexes, leads to product distributions similar to those observed with complex **2** either with (toluene) or without solvent (Table 1, entries 13 and 14).

Having obtained the optimal catalytic conditions and the more efficient pre-catalyst **2**, we studied the influence of the steric hindrance of the substrates (Table 2). The following tests were conducted using four arylamines (aniline, *o*-toluidine, *o*-anisidine, and *p*-anisidine) and two arylmethyl alcohols (benzyl alcohol and 2-methoxybenzyl alcohol).

After 24 h at 120 °C, the reaction between non-sterically hindrance aniline and benzyl alcohol had reached quasi-full conversion. The chemoselectivity against *N*-benzylaniline was 99% (Table 2, entry 1). Repeating the run with *o*-toluidine resulted in a significantly reduced conversion, with only 17% of *o*-toluidine converted. The chemoselectivity of the reaction was slightly affected, as shown in entry 2 of Table 2. Surprisingly, the substitution of benzyl alcohol with the more sterically hindrance 2-methoxybenzyl alcohol increased the rate of the reaction to a conversion of 24% (Table 2, entry 3). The use of methoxy (positive mesomeric effect +M) instead of methyl (inductive electron donor effect +I) as the substituent on the aniline greatly increased the reactivity of the catalytic system; in this case, a conversion of 64% was observed (Table 2, entry 4). As anticipated, modifying the position of the methoxy substituent on the amine aryl substrate (*p*-anisidine) enables near total conversion, regardless of the alcohol used (Table 2, entries 5 and 6).

### 2.4. Mechanistic Computation

The catalytic tests demonstrate that the relative position of the two metal centers on the resorcin[4]arene platform directly affects the rate of the catalytic reaction, as illustrated by the superior performance of complex **2**, where the two ruthenium atoms are attached to two proximal aromatic rings. DFT calculations on the *N*-alkylation of aniline with benzyl alcohol were carried out using complex **3** as the pre-catalyst. This catalytic reaction can be divided into three steps: (i) the activation of the pre-catalyst and the oxidation of benzyl alcohol into benzaldehyde, (ii) the formation of *N*-benzylideneaniline from benzaldehyde and aniline, and (iii) the reduction of the imine into *N*-benzylaniline [30]. As is typically observed with amine or carbonyl function ligands, an outer-sphere pathway is a viable option [26,31].

**Scheme 4:**
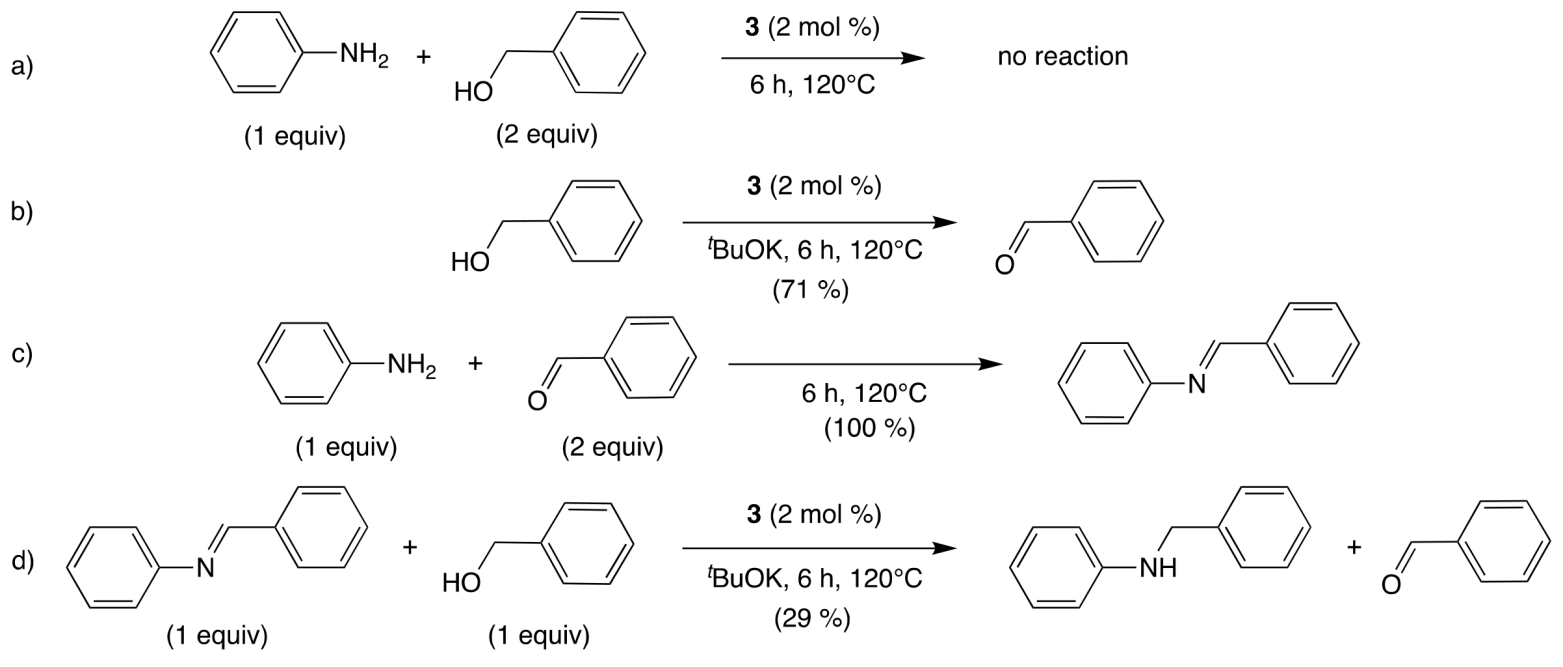
Control experiments.

To validate these three steps, control experiments were carried out using complex **3** (2 mol%) and *^t^*BuOK as a base at 120 °C for 6 h (Scheme 4). As expected, in the absence of a base, no reaction was observed (Scheme 4, Equation a) [28]. The dehydrogenation of benzyl alcohol using the ruthenium catalyst generates benzaldehyde with a conversion of 71% (Scheme 4, Equation b). Subsequently, an excess of benzaldehyde reacts with aniline without catalyst to quantitatively obtain the *N*-benzylideneaniline intermediate (Scheme 4, Equation c). Note that in the presence of ruthenium complex **3**, the full conversion of aniline into imine was also observed. Furthermore, in the presence of benzyl alcohol, the imine is reduced into the *N*-benzylaniline (29% of conversion) with the formation of benzaldehyde (Scheme 4, Equation d).

**Figure 4:**
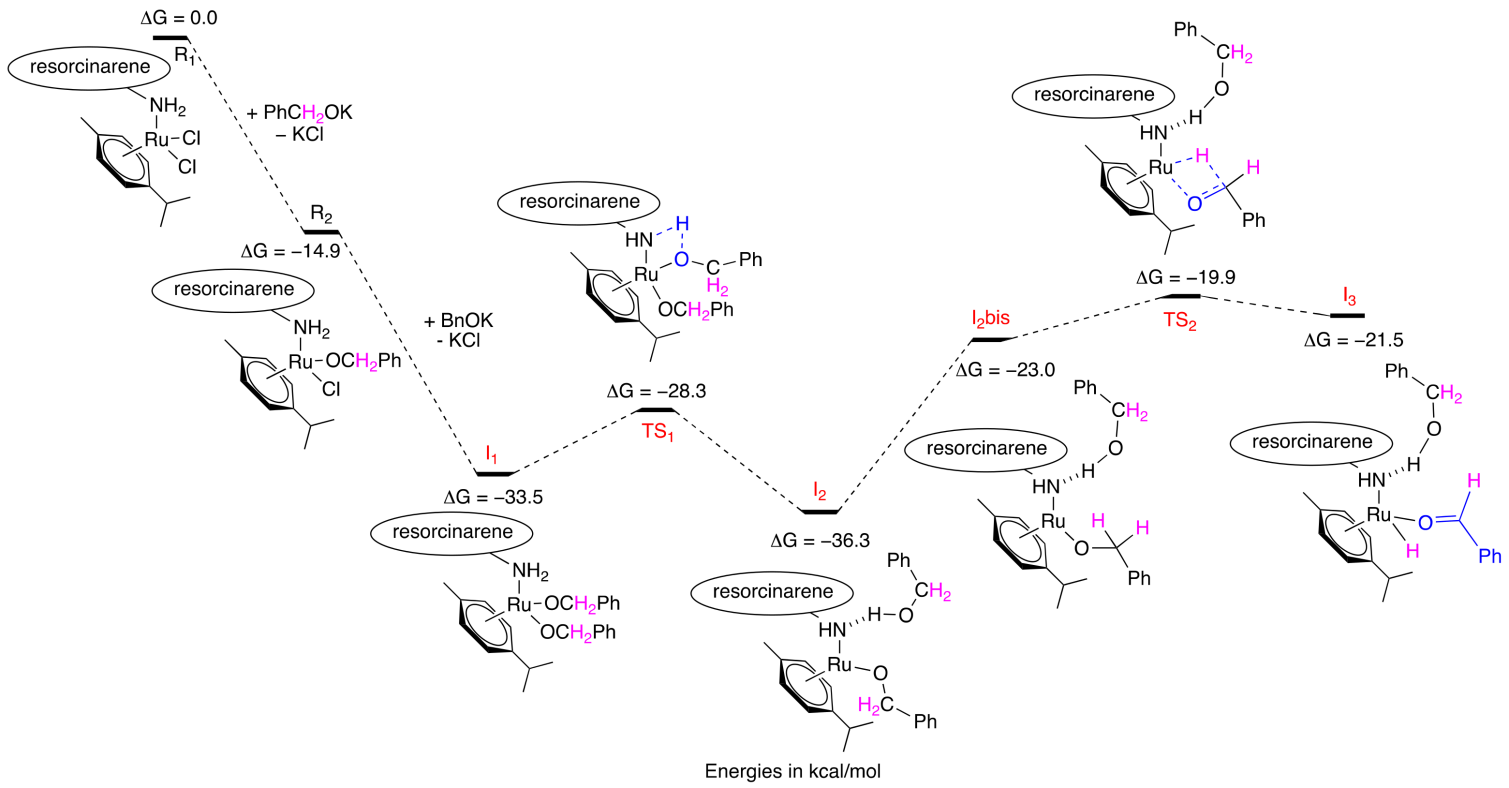
Free energy profiles for the formation of aldehyde (step 1).

In order to clarify the mechanistic aspects, a computational study using the ruthenium complex **3** was carried out. First, we investigated the activation of the ruthenium complex by substituting the two chloride ligands with potassium benzoate. This process occurs in two steps, leading to the more stable intermediate **I_1_** (ΔG = −33.5 kcal/mol). The formation of aldehyde then proceeds via the release of a benzoate ligand as benzyl alcohol, which remains in the metal coordination sphere through an interaction with the amine. The intermediate of ruthenium(II) **I_2_** (ΔG = −36.3 kcal/mol) is generated via the transition state **TS_1_**, in which a proton from the amine is transferred to the benzoate ligand (ΔG = −28.3 kcal/mol). A rearrangement of the coordination sphere of the complex is necessary (less stable **I_2_bis** intermediate, ΔG = −23.0 kcal/mol) before the abstraction of the hydrogen atom of the benzoate ligand to form, via the transition step **TS_2_** (ΔG = −19.9 kcal/mol), the benzaldehyde, which remains coordinated to the ruthenium atom (intermediate **I_3_** with ΔG = −21.5 kcal/mol) (Figure 4).

**Figure 5:**
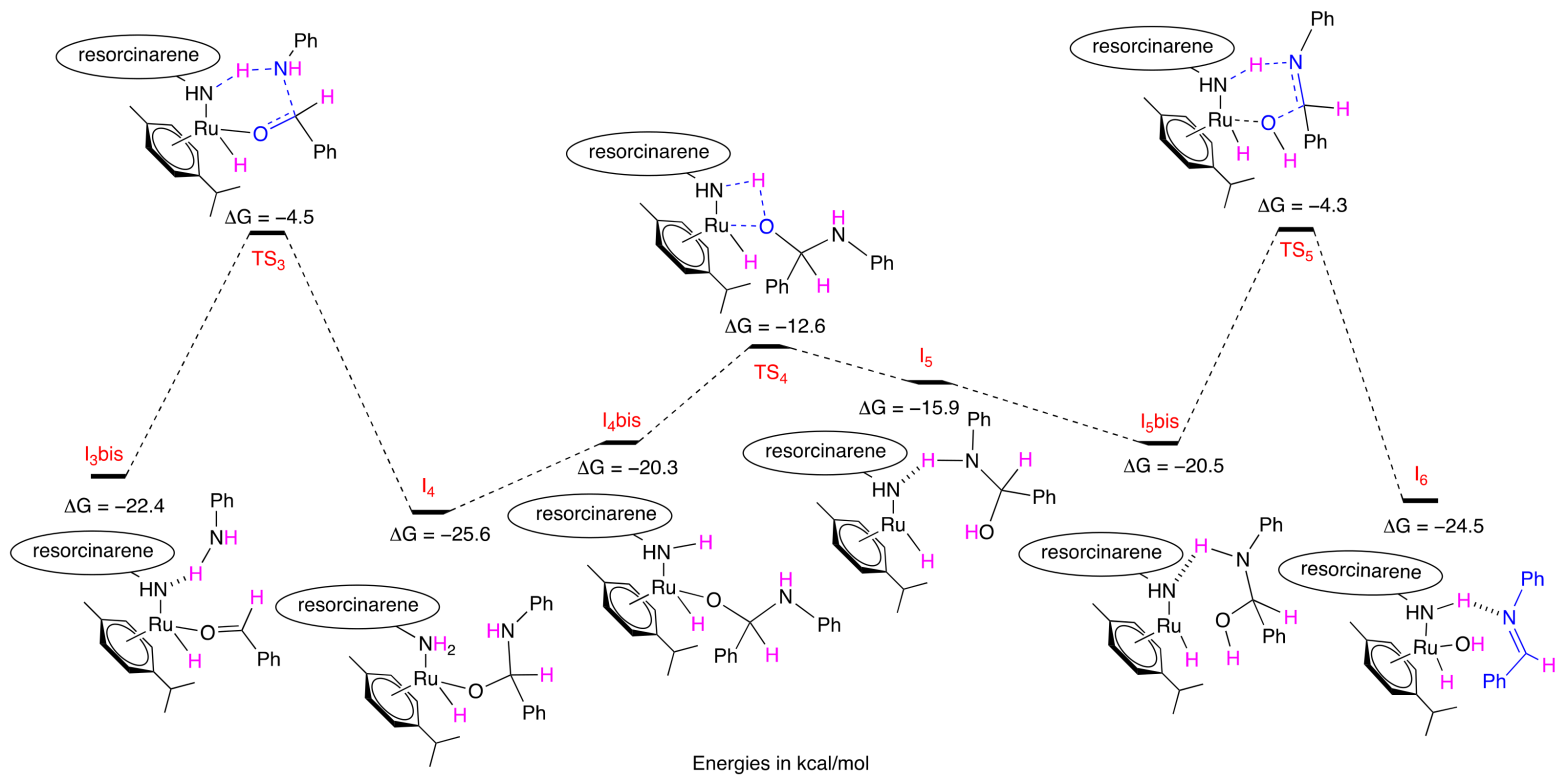
Free energy profiles for the formation of imine (step 2).

After the benzyl alcohol/aniline ligand exchange (intermediate **I_3_bis**), the C-N bond was formed between the amine and the benzaldehyde. This new bond was formed via the 6-membered ring transition state **TS_3_** with a high barrier of ΔG = −4.5 kcal/mol. A proton from the aniline was transferred to the amine of the cavitand. The resulting intermediate **I_4_** (ΔG = −25.6 kcal/mol) was rearranged into **I_4_bis** (ΔG = −20.3 kcal/mol) before the generation of the phenyl(phenylamino)methanol. This step occurred via **TS_4_** (ΔG = −12.6 kcal/mol) and the transfer of a hydrogen atom of the amine to the alcoholate. The formed aminoalcohol was maintained in the coordination sphere of the ruthenium through interactions between the primary amine of cavitand and the hydrogen of the secondary amine of the product (intermediates **I_5_** and **I_5_bis**; ΔG = −15.9 and −20.5 kcal/mol, respectively). The final step was the dehydration of the aminoalcohol, which required the high-energy transition state **TS_7_** (ΔG = −4.3 kcal/mol), in which a hydrogen atom and the hydroxyl moiety were transferred to the amines of the cavitand and the ruthenium atom, respectively. The resulting imine remained in the coordination sphere of the metal through interaction between the macrocyclic amine and the nitrogen atom of the imine (**I_6_**, ΔG = −24.5 kcal/mol) (Figure 5).

**Figure 6:**
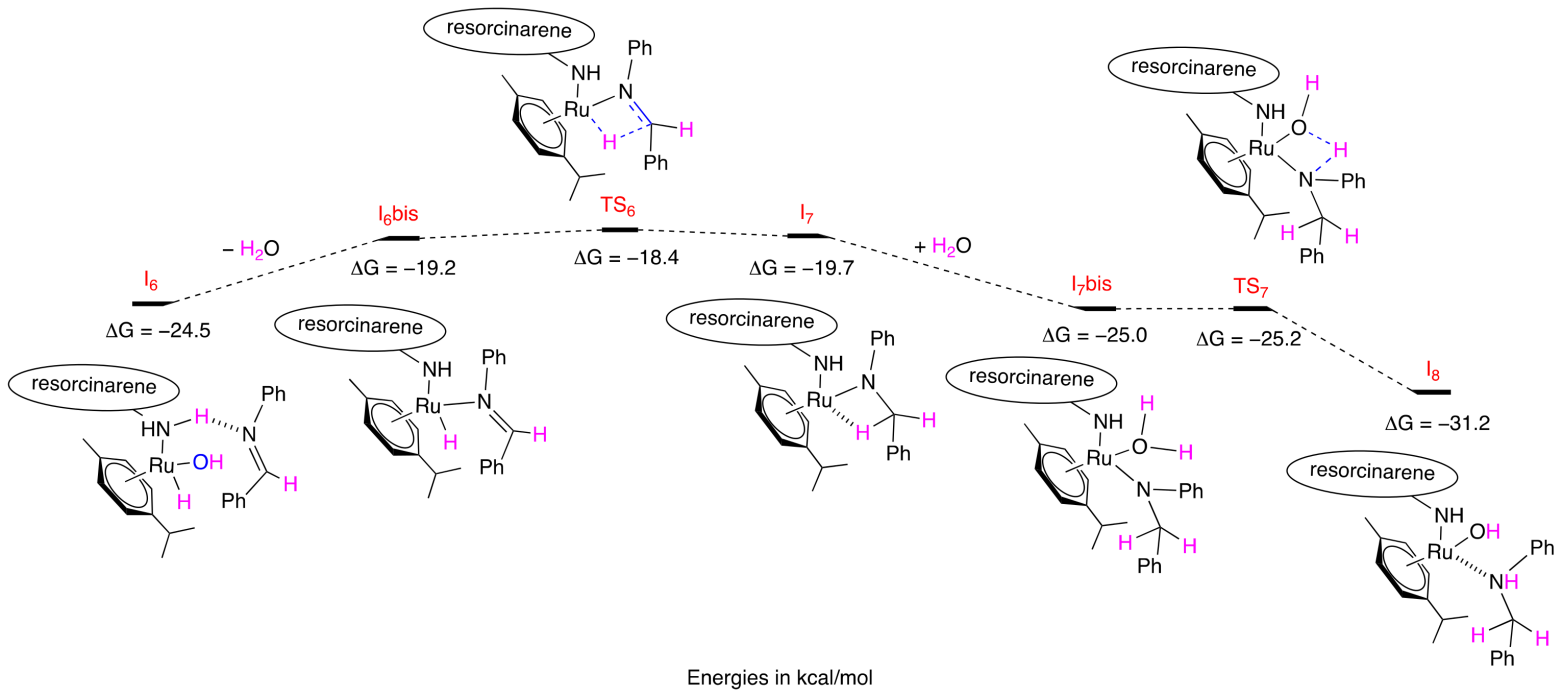
Free energy profiles for the formation of amine (step 3).

The reduction of imine into amine occurred via the elimination of H_2_O and the coordination of the imine as an L-type ligand to the ruthenium atom (intermediate **I_6_bis**, ΔG = −19.2 kcal/mol). The formation of the C-H bond results from the breaking of the Ru-H bond in **TS_6_** (ΔG = −18.4 kcal/mol) creating intermediate **I_7_** (ΔG = −19.7 kcal/mol). In this intermediate, the nitrogen atom of the substrate is coordinated to the metal as a X-type ligand. The coordination of the H_2_O molecule to the ruthenium atom (**I_7_bis**, ΔG = −25.0 kcal/mol) allowed the formation of the N-H bond of the amine product via the low-energy transition state **TS_7_** (ΔG = −25.2 kcal/mol). The resulting catalytic product was kept in the coordination sphere of the ruthenium atom (intermediate **I_8_**, ΔG = −31.2 kcal/mol) (Figure 6).

**Figure 7:**
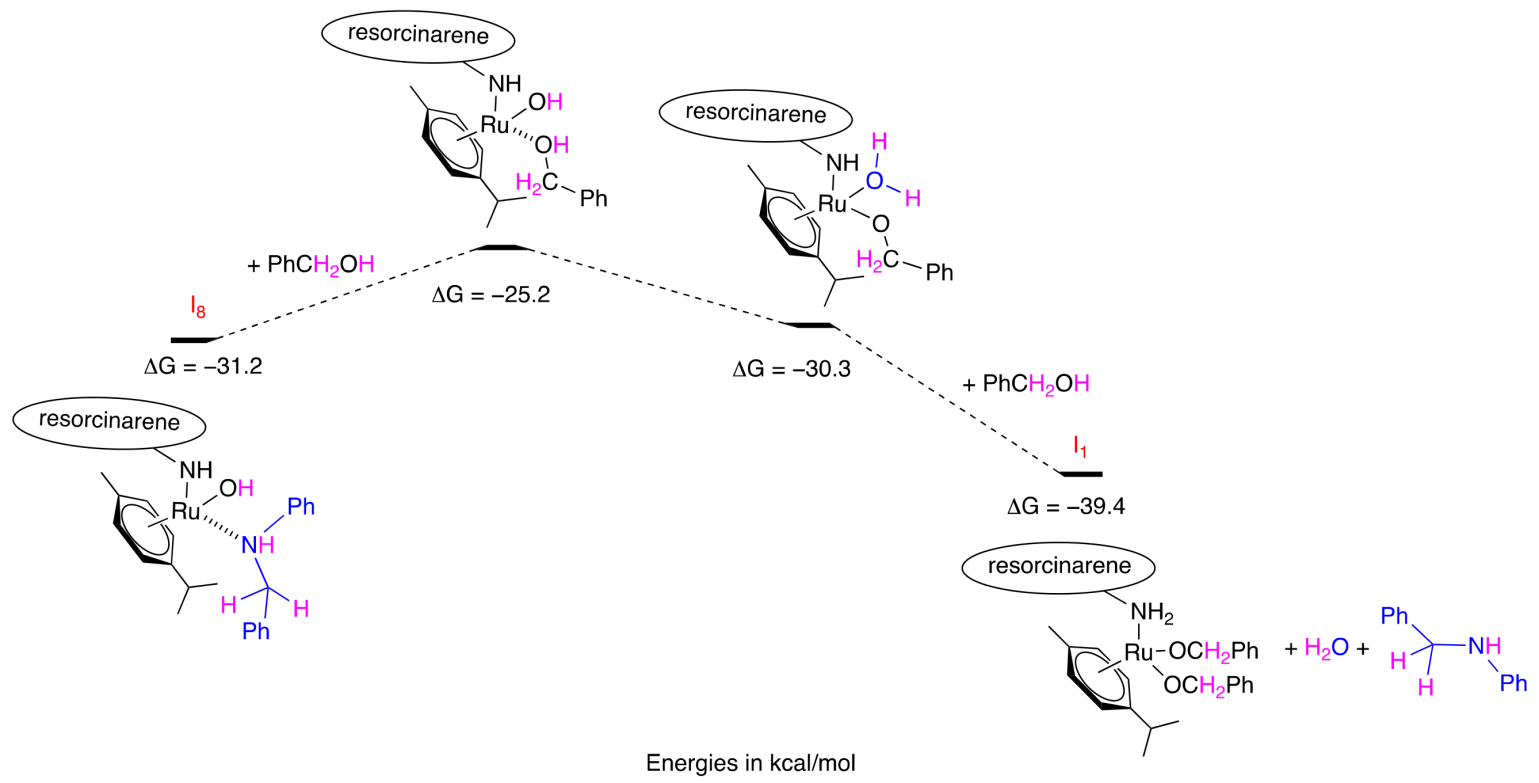
Free energy profiles for the regeneration of the active species.

Finally, starting from the intermediate **I_8_**, it is possible to regenerate the active species **I_1_** via a series of ligand exchanges and the transfer of one proton from the benzyl alcohol to the amine of the macrocycle (Figure 7). This sequence is exothermic with the final structure, which comprises the formation of water molecule and the release amine at −39.4 kcal/mol (slightly more stable than **I_1_** in Figure 4). It should be noted that in the fullly computed mechanism, the ruthenium cation remains at the oxidative state of +II.

The comparison of catalytic systems (complexes **1**–**3** in Table 1, entries 9–11) irrefutably demonstrates that the most effective system is the one where the two metal centers are grafted on proximal aromatic rings of the resorcinarenyl preorganization platform (complex **2**). It is notable that the second bis-ruthenium complex **1**, in which the metals are positioned on the distal aromatics of the macrocycle, results in a catalytic system that is almost identical to that of the mono-metal pre-catalyst **3**. This latter comparison seems to indicate that for complex **1**, resorcinarene is substituted by two totally independent active centers, similar to the mono-ruthenium complex **3**.

**Figure 8:**
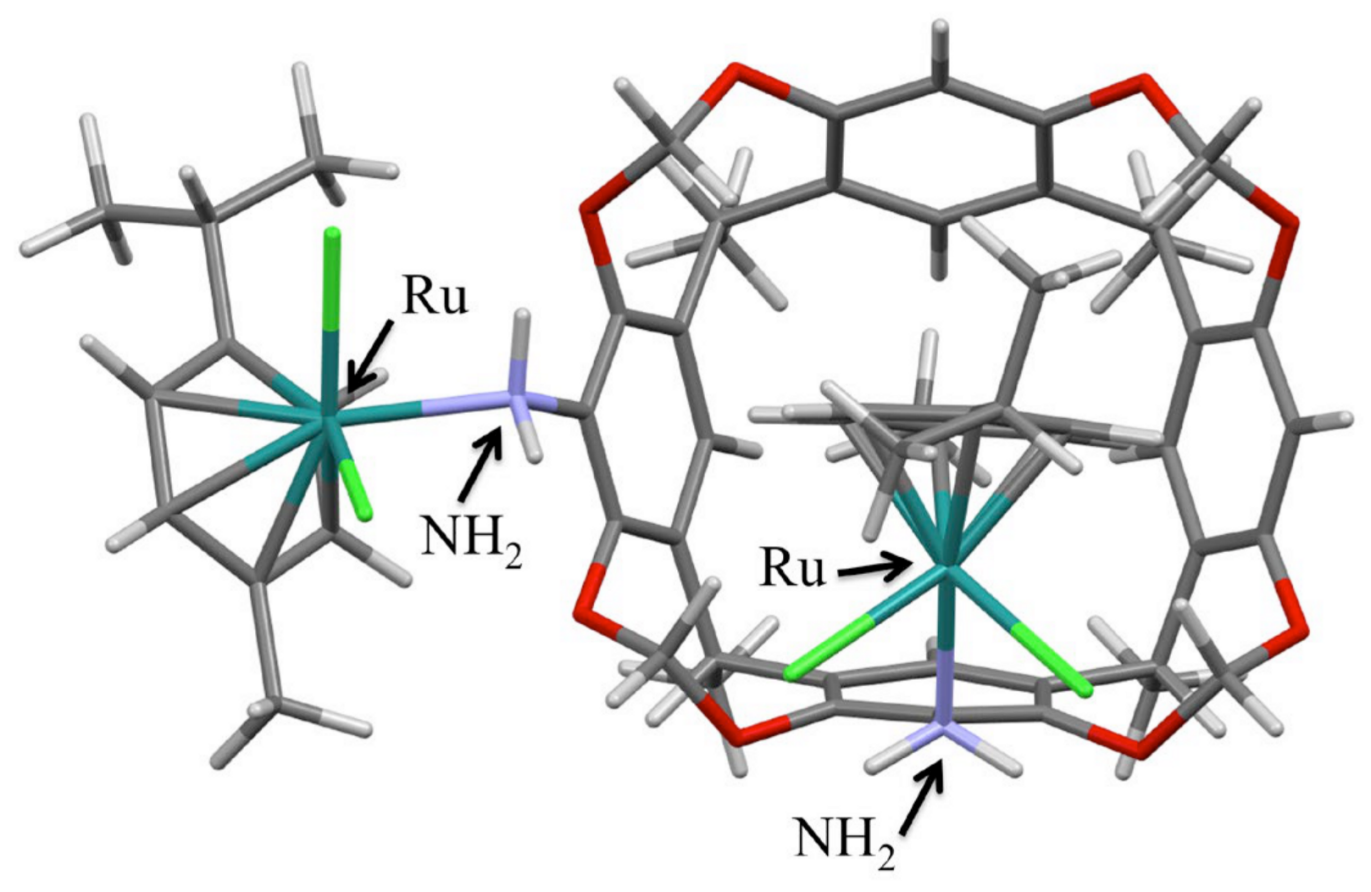
Simulation of complex **2** with ruthenium atoms (in green) adopting an octahedral coordination geometry.

The simulation of complex **2** shows that this molecule is more stable when one ruthenium atom is oriented towards the cavity axis of the resorcin[4]arene, with the second atom adopting an *exo*-orientation (ΔG = G(*exo*-Ru)-G(*endo*-Ru) = −3.4 kcal/mol; Figure 8). The *endo*-orientation of the ruthenium complex was maintained thanks to CH•••π interactions between the CH of the *p*-cymene ligand and aromatic rings of the macrocycle (H•••C lengths in the range 2.618 to 3.659 Å) [32]. With the *p*-cymene ligand trapped in the resorcinarenic cavity, the distance between the *endo*-oriented ruthenium atom and the NH_2_ moiety coordinated to the *exo*-oriented ruthenium is 5.811 Å, which is close enough to permit cooperation between the catalytic center and the amine of the second metallic atom.

Of course, it is quite conceivable that for the complexes **1** and **3**, a “Ru(*p*-cymene)” moiety should be trapped by the resorcinarene cavity, ΔG = G(*exo*-Ru)-G(*endo*-Ru) = −2.0 and −2.5 kcal/mol, respectively, for **1** and **3**. However, the *endo*-orientation of a metal center in complex **1** could not promote a cooperative effect between the *endo*-ruthenium atom and the second NH_2_ moiety, since the distance between the two NH_2_ moieties is too long (9.132 Å).

The theoretical study of the mechanism of *N*-alkylation of aniline with benzylic alcohol requires two transition states high in energy **TS_3_** and **TS_5_** with ΔG = −4.5 and −4.3 kcal/mol, respectively. Given the spatial proximity between the catalytic center and the amine of the second metal, it is reasonable to conclude that an interaction between the latter amine and the nitrogen atom of the aniline (**TS_3_**) and the aminoalcohol (**TS_5_**) is possible. This should decrease the energy of these two transition states, in which the two hydrogen atoms of aniline are transferred to the amine of the cavitand.

Furthermore, the catalytic tests showed that the rate of the reaction is contingent upon the nature of the substituent of aniline. Indeed, the steric hindrance generated by the methyl moiety in *o*-toluidine makes cooperation with the second amine of the cavitand more difficult, with **TS_3_** and **TS_5_** being high in energy. Conversely the presence of a methoxy substituent on aniline (*o*-anisidine) allows for the movement of a proton between the aniline or aminoalcohol and the coordinated amine of the metal center. The energies of the two transition states, **TS_3_** and **TS_5_**, should be lowered or they will be slightly affected by the sterically hindered methoxy group in *ortho* position of the anisidine.

## 3. Materials and Methods

**General Remarks:** All manipulations were carried out in Schlenk-type flasks under dry argon. Solvents were dried by conventional methods and were distilled immediately before use. Routine ^1^H and ^13^C{^1^H} spectra were recorded with AC 300 and 500 Bruker FT instruments. Chemical shifts and coupling constants are reported in ppm and Hz, respectively. ^1^H and ^13^C{^1^H} NMR spectra were recorded in CDCl_3_ or C_6_D_6_ and they referenced residual protonated solvent (*δ* = 7.26 or 7.16 and 77.16 or 128.06 ppm, respectively). Infrared spectra were recorded on a Bruker ATR FT-IR Alpha-P spectrometer. Elemental analyses were carried out by the Service de Microanalyse, Institut de Chimie, Université de Strasbourg. The catalytic solutions were analyzed with a Varian 3900 gas chromatograph fitted with a WCOT fused silica column (25 m × 0.25 mm, 0.25 μm film thickness). 5,17-Dibromo-4(24),6(10),12(16),18(22)-tetramethylenedioxy-2,8,14,20-tetra-pentyl-resorcin[4]arene (**7**) [10], 5,11-dibromo-4(24),6(10),12(16),18(22)-tetramethylene-dioxy-2,8,14,20-tetrapentyl-resorcin[4]arene (**8**) [10], tosyl azide [33], and 5-amino-4(24),6(10),12(16),18(22)-tetramethylenedioxy-2,8,14,20-tetrapentyl-resorcin[4] arene (**6**) [23] were prepared according to an adapted published procedure.

### 3.1. Synthesis

#### 3.1.1. General Procedure for the Synthesis of 5,X-Diazido-4(24),6(10),12(16),18(22)-tetra-methyl-enedioxy-2,8,14,20-tetrapentyl-resorcin[4]arene (X = 11 or 17)

*n*-Butyllithium (1.6 M in hexane; 4.96 mL, 7.94 mmol) was slowly added to a solution of dibromo-cavitand (3.000 g, 3.31 mmol) in THF (150 mL) at −78 °C. After 1 h, the resulting anion was quenched with tosyl azide (1.560 g, 7.94 mmol) and the mixture was stirred at room temperature for 24 h. The reaction mixture was washed three times with brine (3 × 100 mL). The solvent was evaporated under reduced pressure, and the crude product was purified by column chromatography (Et_2_O/petroleum ether).

**5,17-Diazido-4(24),6(10),12(16),18(22)-tetramethylenedioxy-2,8,14,20-tetrapentyl-resorcin[4]arene (9):** Yield: 2.973 g (84%). FT-IR: ν(N_3_) 2102 and 1283 cm^−1^. ^1^H NMR (500 MHz, CDCl_3_): ^1^H NMR (300 MHz, CDCl_3_): *δ* = 7.08 (s, 2H, arom. CH), 6.87 (s, 2H, arom. CH), 6.55 (s, 2H, arom. CH), 5.79 and 4.42 (AB spin system, 8H, OCH_2_O, ^2^*J_HH_* = 7.2 Hz), 4.73 (t, 4H, C*H*CH_2_, ^3^*J_HH_* = 8.1 Hz), 2.25–2.14 (m, 8H, CHC*H_2_*), 1.44–1.31 (m, 24H C*H_2_*C*H_2_*C*H_2_*CH_3_), 0.92 (t, 12H, CH_2_C*H_3_*, ^3^*J_HH_* = 7.0 Hz); ^13^C{^1^H} NMR (126 MHz, CDCl_3_): *δ* = 155.11–115.44 (arom. C’s)_,_ 100.05 (s, OCH_2_O), 36.75 (s, *C*HCH_2_), 32.10 (s, *C*H_2_CH_2_CH_3_), 29.90 (s, CH*C*H_2_), 27.65 (s, CHCH_2_*C*H_2_), 22.82 (s, *C*H_2_CH_3_), 14.23 (s, CH_2_*C*H_3_) ppm. Elemental analysis (%): calcd for C_52_H_62_O_8_N_6_ (899.08): C: 69.47; H: 6.95; N: 9.35; found C: 69.62; H: 7.04 N: 9.28.

**5,11-Diazido-4(24),6(10),12(16),18(22)-tetramethylenedioxy-2,8,14,20-tetrapentyl-resorcin[4]arene (10):** Yield: 2.432 g (82%). FT-IR: ν(N_3_) 2103 and 1285 cm^−1^. ^1^H NMR (300 MHz, CDCl_3_): *δ* = 7.09 (s, 2H, arom. CH), 6.87 (s, 2H, arom. CH), 6.51 (s, 2H, arom. CH), 5.81 and 4.42 (AB spin system, 2H, OCH_2_O, ^2^*J_HH_* = 6.9 Hz), 5.79 and 4.42 (AB spin system, 4H, OCH_2_O, ^2^*J_HH_* = 6.9 Hz), 5.77 and 4.40 (AB spin system, 2H, OCH_2_O, ^2^*J_HH_* = 7.2 Hz), 4.73 (t, 4H, C*H*CH_2_, ^3^*J_HH_* = 8.1 Hz), 2.24–2.14 (m, 8H, CHC*H_2_*), 1.45–1.30 (m, 24H C*H_2_*C*H_2_*C*H_2_*CH_3_), 0.92 (t, 12H, CH_2_C*H_3_*, ^3^*J_HH_* = 6.9 Hz); ^13^C{^1^H} NMR (126 MHz, CDCl_3_): *δ* = 155.15–115.67 (arom. C’s)_,_ 100.18 (s, OCH_2_O), 100.02 (s, OCH_2_O), 99.78 (s, OCH_2_O), 37.00 (s, *C*HCH_2_), 36.75 (s, *C*HCH_2_), 36.50 (s, *C*HCH_2_), 32.14 (s, *C*H_2_CH_2_CH_3_), 32.10 (s, *C*H_2_CH_2_CH_3_), 32.06 (s, *C*H_2_CH_2_CH_3_), 29.95 (s, CH*C*H_2_), 29.91 (s, CH*C*H_2_), 29.86 (s, CH*C*H_2_), 27.67 (s, CHCH_2_*C*H_2_), 27.65 (s, CHCH_2_*C*H_2_), 27.62 (s, CHCH_2_*C*H_2_), 22.83 (s, *C*H_2_CH_3_), 22.80 (s, *C*H_2_CH_3_), 22.79 (s, *C*H_2_CH_3_), 14.27 (s, CH_2_*C*H_3_), 14.23 (s, CH_2_*C*H_3_) ppm. Elemental analysis (%): calcd for C_52_H_62_O_8_N_6_ (899.08): C: 69.47; H: 6.95; N: 9.35; found C: 69.55; H: 7.02 N: 9.31.

#### 3.1.2. General Procedure for the Synthesis of 5,X-Diamino-4(24),6(10),12(16),18(22)-te-tramethylenedioxy-2,8,14,20-tetrapentyl-resorcin[4]arene (X = 11 or 17)

The hydrogenation experiment was carried out in a glass-lined stainless steel autoclave (100 mL) containing a magnetic stirrer bar. Palladium-on-carbon (10% Pd *w*/*w*, 0.200 g) was added. The autoclave was closed, flushed twice with hydrogen, a solution of diazide-cavitand (1.500 g, 1.66 mmol) in CH_2_Cl_2_ (15 mL) was added, the autoclave pressurized to 5 bar, and the reaction mixture was stirred at room temperature for 24 h. The autoclave was depressurized, the solution was passed through a plug of Celite, and the solvent was evaporated under reduced pressure.

**5,17-Diamino-4(24),6(10),12(16),18(22)-tetramethylenedioxy-2,8,14,20-tetrapentyl-resorcin[4]arene (4):** Yield 1.395 g (100%). ^1^H NMR (300 MHz, CDCl_3_): *δ* = 7.08 (s, 2H, arom. CH), 6.48 (s, 2H, arom. CH), 6.47 (s, 2H, arom. CH), 5.79 and 4.42 (AB spin system, 8H, OCH_2_O, ^2^*J_HH_* = 6.9 Hz), 4.68 (t, 4H, C*H*CH_2_, ^3^*J_HH_* = 8.1 Hz), 3.77 (s, 4H, NH_2_), 2.24–2.12 (m, 8H, CHC*H_2_*), 1.44–1.31 (m, 24H C*H_2_*C*H_2_*C*H_2_*CH_3_), 0.91 (t, 12H, CH_2_C*H_3_*, ^3^*J_HH_* = 7.0 Hz); ^13^C{^1^H} NMR (126 MHz, CDCl_3_): *δ* = 154.80–107.32 (arom. C’s)_,_ 99.28 (s, OCH_2_O), 36.77 (s, *C*HCH_2_), 32.23 (s, *C*H_2_CH_2_CH_3_), 29.93 (s, CH*C*H_2_), 27.79 (s, CHCH_2_*C*H_2_), 22.85 (s, *C*H_2_CH_3_), 14.27 (s, CH_2_*C*H_3_) ppm. Elemental analysis (%): calcd for C_52_H_66_O_8_N_2_ (847.09): C: 73.73; H: 7.85; N: 3.31; found C: 73.67; H: 7.78 N: 3.24.

**5,11-Diamino-4(24),6(10),12(16),18(22)-tetramethylenedioxy-2,8,14,20-tetrapentyl-resorcin[4]arene (5):** Yield: 1.412 g (100%). ^1^H NMR (300 MHz, CDCl_3_): *δ* = 7.09 (s, 2H, arom. CH), 6.50 (s, 2H, arom. CH), 6.48 (s, 2H, arom. CH), 5.84 and 4.32 (AB spin system, 2H, OCH_2_O, ^2^*J_HH_* = 6.9 Hz), 5.79 and 4.41 (AB spin system, 4H, OCH_2_O, ^2^*J_HH_* = 6.9 Hz), 5.74 and 4.51 (AB spin system, 2H, OCH_2_O, ^2^*J_HH_* = 7.2 Hz), 4.71 (t, 1H, C*H*CH_2_, ^3^*J_HH_* = 8.4 Hz), 4.68 (t, 2H, C*H*CH_2_, ^3^*J_HH_* = 8.1 Hz), 4.65 (t, 1H, C*H*CH_2_, ^3^*J_HH_* = 8.1 Hz), 3.75 (s br, 4H NH_2_), 2.25–2.11 (m, 8H, CHC*H_2_*), 1.44–1.31 (m, 24H C*H_2_*C*H_2_*C*H_2_*CH_3_), 0.91 (t, 12H, CH_2_C*H_3_*, ^3^*J_HH_* = 7.2 Hz); ^13^C{^1^H} NMR (126 MHz, CDCl_3_): *δ* = 154.97–107.77 (arom. C’s)_,_ 99.61 (s, OCH_2_O), 99.29 (s, OCH_2_O), 98.95 (s, OCH_2_O), 37.02 (s, *C*HCH_2_), 36.77 (s, *C*HCH_2_), 36.51 (s, *C*HCH_2_), 32.27 (s, *C*H_2_CH_2_CH_3_), 32.23 (s, *C*H_2_CH_2_CH_3_), 32.18 (s, *C*H_2_CH_2_CH_3_), 30.02 (s, CH*C*H_2_), 29.93 (s, CH*C*H_2_), 29.82 (s, CH*C*H_2_), 27.84 (s, CHCH_2_*C*H_2_), 27.79 (s, CHCH_2_*C*H_2_), 27.73 (s, CHCH_2_*C*H_2_), 22.86 (s, *C*H_2_CH_3_), 22.85 (s, *C*H_2_CH_3_), 22.84 (s, *C*H_2_CH_3_), 14.28 (s, CH_2_*C*H_3_), 14.26 (s, CH_2_*C*H_3_), 14.25 (s, CH_2_*C*H_3_) ppm. Elemental analysis (%): calcd for C_52_H_66_O_8_N_2_ (847.09): C: 73.73; H: 7.85; N: 3.31; found C: 73.62; H: 7.74 N: 3.26.

#### 3.1.3. General Procedure for the Synthesis of Ruthenium Complexes (**1**–**3**)

A solution of [RuCl_2_(*p*-cymene)_2_]_2_ (0.5 or 1 equiv./amino-cavitand) in CH_2_Cl_2_ (10 mL) was added to a stirred solution (CH_2_Cl_2_, 5 mL) of amino-cavitand (0.36 mmol) and was stirred at room temperature for 24 h. The reaction mixture was concentrated at ca. 1 mL, after which Et_2_O (50 mL) was added. The red precipitate was separated by filtration and dried under vacuum.

***N*,*N′*-{5,17-Diamino-4(24),6(10),12(16),18(22)-tetramethylenedioxy-2,8,14,20-tetra-pentylresorcin[4]arene}-bis-[dichloro-(*p*-cymene)-ruthenium(II)] (1):** Yield: 0.362 g (71%). ^1^H NMR (300 MHz, CDCl_3_): *δ* = 7.08 (s, 2H, arom. CH of resorcinarene), 6.97 (s, 2H, arom. CH of resorcinarene), 6.59 (s, 2H, arom. CH of resorcinarene), 29 (br s, 1H, arom. CH of resorcinarene), 5.93 (A part of the AB spin system, 4H, OCH_2_O, ^2^*J_HH_* = 6.3 Hz), 5.47 and 5.34 (AA’BB’ spin system, 4H, arom. CH of *p*-cymene, ^3^*J_HH_* = 6.0 Hz), 4.84–4.55 (m, 16H, arom. CH of *p*-cymene, B part of the AB spin system of OCH_2_O, NH_2_ and C*H*CH_2_), 2.94–2.83 (m, 2H, C*H*(CH_3_)_2_), 2.44–2.30 (m, 4H, CHC*H_2_*CH_2_), 2.23–2.13 (m, 4H, CHC*H_2_*CH_2_), 2.19 (s, 3H, CH_3_ of *p*-cymene), 2.15 (s, 3H, CH_3_ of *p*-cymene), 1.48–1.32 (m, 24H *CH_2_CH_2_CH_2_*CH_3_), 1.27 (d, 6H, CH(C*H*_3_)_2_, ^3^*J_HH_* = 7.2 Hz), 1.24 (d, 6H, CH(C*H*_3_)_2_, ^3^*J_HH_* = 6.6 Hz), 0.91 (t, 12H, CH_2_*CH_3_*, ^3^*J_HH_* = 6.9 Hz); ^13^C{^1^H} NMR (126 MHz, CDCl_3_): *δ* = 154.86–115.11 (arom. C’s), 104.82 (s, C_quart._ of *p*-cymene), 101.37 (s, C_quart._ of *p*-cymene), 100.28 (s, OCH_2_O), 96.89 (s, C_quart._ of *p*-cymene), 94.11 (s, C_quart._ of *p*-cymene), 83.44 (s, arom. CH of *p*-cymene), 81.45 (s, arom. CH of *p*-cymene), 80.68 (s, arom. CH of *p*-cymene), 77.99 (s, arom. CH of *p*-cymene), 36.63 (s, *C*HCH_2_), 31.98 (s, *C*H_2_CH_2_CH_3_), 30.76 (s, *C*H(CH_3_)_2_), 30.55 (s, *C*H(CH_3_)_2_), 29.86 (s, CH*C*H_2_), 27.77 (s, CHCH_2_*C*H_2_), 22.89 (s, *C*H_2_CH_3_), 22.28 (s, CH(*C*H_3_)_2_), 22.03 (s, CH(*C*H_3_)_2_), 19.06 (s, CH_3_ of *p*-cymene), 18.43 (s, CH_3_ of *p*-cymene), 14.24 (s, CH_2_*C*H_3_) ppm. MS (ESI-TOF): *m*/*z* = 1423.42 [M − Cl]^+^ (expected isotopic profile). Elemental analysis (%): calcd for C_72_H_94_O_8_N_2_Cl_4_Ru_2_ (1459.47): C: 59.25; H: 6.49; N: 1.92; found C: 59.37; H: 6.55 N: 1.87.

***N*,*N′*-{5,11-Diamino-4(24),6(10),12(16),18(22)-tetramethylenedioxy-2,8,14,20-tetra-pentylresorcin[4]arene}-bis-[dichloro-(*p*-cymene)-ruthenium(II)] (2):** Yield: 0.347 g (68%). ^1^H NMR (300 MHz, CDCl_3_): *δ* = 7.10 (s, 2H, arom. CH of resorcinarene), 6.97 (br s, 2H, arom. CH of resorcinarene), 6.53 (s, 2H, arom. CH of resorcinarene), 6.09–5.47 (m, 3H, A part of the AB spin system of OCH_2_O), 5.73 (A part of the AB spin system, 1H, OCH_2_O, ^2^*J_HH_* = 6.9 Hz), 5.48 and 5.34 (AA’BB’ spin system, 8H, arom. CH of *p*-cymene, ^3^*J_HH_* = 6.0 Hz), 4.85–4.30 (m, 12H, B part of the AB spin system of OCH_2_O, NH_2_ and C*H*CH_2_), 2.97–2.83 (m, 2H, C*H*(CH_3_)_2_), 2.41–2.18 (m, 8H, CHC*H_2_*CH_2_), 2.17 (s, 3H, CH_3_ of *p*-cymene), 2.16 (s, 3H, CH_3_ of *p*-cymene), 1.58–1.31 (m, 24H *CH_2_CH_2_CH_2_*CH_3_), 1.28 (d, 12H, CH(C*H*_3_)_2_, ^3^*J_HH_* = 6.9 Hz), 0.92 (t, 9H, CH_2_*CH_3_*, ^3^*J_HH_* = 7.0 Hz), 0.85 (t, 3H, CH_2_*CH_3_*, ^3^*J_HH_* = 7.2 Hz) ppm; ^13^C{^1^H} NMR (126 MHz, C_6_D_6_): *δ* = 155.70–117.26 (arom. C’s), 100.34 (s, C_quart._ of *p*-cymene), 99.63 (s, OCH_2_O), 99.47 (s, OCH_2_O), 99.46 (s, OCH_2_O), 96.37 (s, C_quart._ of *p*-cymene), 81.19 (s, arom. CH of *p*-cymene), 80.48 (s, arom. CH of *p*-cymene), 37.36 (s, *C*HCH_2_), 37.17 (s, *C*HCH_2_), 36.96 (s, *C*HCH_2_), 32.32 (s, *C*H_2_CH_2_CH_3_), 32.29 (s, *C*H_2_CH_2_CH_3_), 30.82 (s, *C*H(CH_3_)_2_), 30.41 (s, CH*C*H_2_), 30.37 (s, CH*C*H_2_), 30.30 (s, CH*C*H_2_), 28.17 (s, CHCH_2_*C*H_2_), 28.09 (s, CHCH_2_*C*H_2_), 27.98 (s, CHCH_2_*C*H_2_), 23.15 (s, *C*H_2_CH_3_), 23.10 (s, *C*H_2_CH_3_), 23.05 (s, *C*H_2_CH_3_), 22.11 (s, CH(*C*H_3_)_2_), 18.82 (s, CH_3_ of *p*-cymene), 14.29 (s, CH_2_*C*H_3_) ppm. MS (ESI-TOF): *m*/*z* = 1458.38 [M]^+^ and 1423.42 [M − Cl]^+^ (expected isotopic profile). Elemental analysis (%): calcd for C_72_H_94_O_8_N_2_Cl_4_Ru_2_ (1459.47): C: 59.25; H: 6.49; N: 1.92; found C: 59.12; H: 6.28 N: 1.85.

***N*-{5-Amino-4(24),6(10),12(16),18(22)-tetramethylenedioxy-2,8,14,20-tetrapentyl-re-sorcin[4]arene}-[dichloro-(*p*-cymene)-ruthenium(II)] (3):** Yield: 0.295 g (72%). ^1^H NMR (300 MHz, C_6_D_6_): *δ* = 7.53 (s, 1H, arom. CH of resorcinarene), 7.49 (s, 2H, arom. CH of resorcinarene), 6.96 (s, 1H, arom. CH of resorcinarene), 6.69 (s, 1H, arom. CH of resorcinarene), 6.36 (s, 2H, arom. CH of resorcinarene), 5.58 and 4.38 (AB spin system, 4H, OCH_2_O, ^2^*J_HH_* = 7.2 Hz), 5.58 and 4.31 (AB spin system, 4H, OCH_2_O, ^2^*J_HH_* = 6.9 Hz), 5.15 (t, 2H, C*H*CH_2_, ^3^*J_HH_* = 7.8 Hz), 5.13 (t, 2H, C*H*CH_2_, ^3^*J_HH_* = 7.8 Hz), 5.01 and 4.83 (AA’BB’ spin system, 4H, arom. CH of *p*-cymene, ^3^*J* = 5.7 Hz), 3.55 (s br, 2H NH_2_), 2.84 (hept, 1H, C*H*(CH_3_)_2_, ^3^*J_HH_* = 6.9 Hz), 2.40–2.23 (m, 8H, CHC*H_2_*CH_2_), 1.83 (s, 3H, CH_3_ of *p*-cymene), 1.42–1.20 (m, 24H *CH_2_CH_2_CH_2_*CH_3_)_,_ 1.04 (d, 6H, CH(C*H*_3_)_2_, ^3^*J_HH_* = 6.9 Hz), 0.89 (t, 3H, CH_2_*CH_3_*, ^3^*J_HH_* = 6.6 Hz), 0.83 (t, 9H, CH_2_*CH_3_*, ^3^*J_HH_* = 7.0 Hz); ^13^C{^1^H} NMR (126 MHz, C_6_D_6_): *δ* = 155.63–116.96 (arom. C’s), 100.27 (s, arom. C_quart._ of *p*-cymene), 99.45 (s, OCH_2_O), 99.20 (s, OCH_2_O), 96.38 (s, arom. C_quart._ of *p*-cymene), 81.19 (s, arom. CH of *p*-cymene), 80.45 (s, arom. CH of *p*-cymene), 37.19 (s, *C*HCH_2_), 36.97 (s, *C*HCH_2_), 32.34 (s, *C*H_2_CH_2_CH_3_), 32.30 (s, *C*H_2_CH_2_CH_3_), 31.96 (s, *C*H_2_CH_2_CH_3_), 30.81 (s, *C*H(CH_3_)_2_), 30.39 (s, *C*H(CH_3_)_2_), 28.08 (s, CHCH_2_*C*H_2_), 27.98 (s, CHCH_2_*C*H_2_), 23.08 (s, *C*H_2_CH_3_), 23.04 (s, *C*H_2_CH_3_), 22.10 (s, CH(*C*H_3_)_2_), 18.86 (s, CH_3_ of *p*-cymene), 14.28 (s, CH_2_*C*H_3_), 14.27 (s, CH_2_*C*H_3_) ppm. MS (ESI-TOF): *m*/*z* = 1138.43 [M + H]^+^ and 1102.45 [M − Cl]^+^ (expected isotopic profile). Elemental analysis (%): calcd for C_62_H_79_O_8_NCl_2_Ru (1138.27): C: 65.42; H: 7.00; N: 1.23; found C: 65.37; H: 6.94 N: 1.19.

### 3.2. X-Ray Crystal Structure Analysis

Single crystals of ruthenium(II) complex **3**, suitable for X-ray analysis, were obtained through the slow diffusion of Et_2_O into a CHCl_3_ solution of the complex. The samples were studied on a Bruker PHOTON-III CPAD, using Mo-K*α* radiation (*λ* = 0.71073 Å) at T = 120(2) K. The structures were solved with SHELXT-2018/2 [34], which revealed the non-hydrogen atoms of the molecule. After anisotropic refinement, all of the hydrogen atoms were found with a Fourier difference map. The structure was refined with SHELXL-2019/3 [35] using the full-matrix least-square technique (use of F square magnitude; x, y, z, and βij for C, Cl, N, O, and Ru atoms; x, y, and z in riding mode for H atoms) (Table 3). CCDC 2409059 contains the supplementary crystallographic data for this paper. The data can be obtained free of charge from the Cambridge Crystallographic Data Centre via www.ccdc.cam.ac.uk/structures (accessed on 13 January 2024).

### 3.3. Catalytic Tests

In a Schlenk tube in an argon atmosphere, a solvent free solution of aniline (1.0 mmol), alcohol (2 mmol), base (1.0 mmol), and ruthenium catalyst (0.02 mmol, 2 mol%, based on ruthenium atom) was stirred at 120 °C for 24 h. After cooling to room temperature, the reaction mixture was diluted with CH_2_Cl_2_ (2 mL) and passed through a Millipore filter and analyzed by GC. All products were unambiguously identified by NMR spectroscopy (^1^H and ^13^C{^1^H}) after their isolation.

### 3.4. Computational Details

All calculations were performed with GAUSSIAN 09 (version D01) [36] at the DFT level of theory with a ωB97XD functional [37]. All atoms except the ruthenium were described by the 6-31+G** basis set [38]. The metal cation was described by the SDD pseudopotential and associated basis set [39]. All calculations were performed in solution using a PCM for aniline [40]. All structures were fully optimized, and the nature of the encountered stationary point was determined by a frequency calculation. Minima were characterized by a full set of real frequencies and the transition state by one imaginary frequency. Gibbs free energies were extracted from this calculation.

## 4. Conclusions

Here, we described the synthesis of three ruthenium(II) complexes, in which one or two “RuCl_2_(*p*-cymene)NH_2_” moieties were grafted onto the upper rim of resorcin[4]arene. The catalytic results of the *N*-alkylation of primary amines with arylmethyl alcohol using the green “hydrogen borrowing” methodology clearly demonstrated that the rate of the catalytic reaction is more important when bis-metallic pre-catalysts are employed, especially when the two metal centers were grafted onto proximal aromatic rings of the cavitand. The proximity between the ruthenium atom and the amine of the secondary grafted metallic center unquestionably explains the superior performance of this catalyst through a decrease in the two transition states higher in energies calculated for the hydrogen atoms transferred from the aniline and the aminoalcohol to the amine linked to the ruthenium. Future work will focus on the development of the resorcin[4]arene as a preorganization platform to study the synergy between a catalytic center and an amine function in a carbon–heteroatom bond formation.

## Figures and Tables

**Table 1:** Ruthenium-catalyzed *N*-alkylation of aniline, search of the optimal conditions ^1^.

Entry	Ruthenium Complex	Base	Time (h)	Solvent	Conversion ^2^ (%)	Product Distribution ^2^ (Amine 14/Imine 13)
1	**3**	*^t^*BuOK	3	toluene	88	6/94
2	**3**	*^t^*BuOK	3	DMF	49	28/72
3	**3**	*^t^*BuOK	3	dioxane	64	26/74
4	**3**	*^t^*BuOK	3	/	40	92/8
5	**3**	KOH	6	/	41	97/3
6	**3**	K_2_CO_3_	6	/	75	13/87
7	**3**	Cs_2_CO_3_	6	/	61	70/30
8	**3**	NaOAc	6	/	55	16/84
9	**3**	*^t^*BuOK	6	/	60	93/7
10	**1**	*^t^*BuOK	6	/	65	80/10
11	**2**	*^t^*BuOK	6	/	82	98/2
12	**D [27]** ^3^	KOH	6	/	78	100/0
13	**E [28]** ^4^	*^t^*BuOK	24	/	97	99/1
14	**F [29]** ^5^	*^t^*BuOK	24	toluene	91	95/5

^1^ Reaction conditions: [Ru] (2 mol%), benzyl alcohol (2 mmol), aniline (1 mmol), base (1 mmol), solvent if necessary (2 mL), 120 °C. ^2^ The conversions and products distribution were determined by GC. ^3^ Reaction conditions: **D** (2 mol%), benzyl alcohol (1 mmol), aniline (1 mmol), KOH (1 mmol), 110 °C. ^4^ Reaction conditions: **E** (2.5 mol%), benzyl alcohol (1.5 mmol), aniline (1 mmol), base (1 mmol), 120 °C. ^5^ Reaction conditions: **F** (2.5 mol%), benzyl alcohol (1 mmol), aniline (1.1 mmol), base (2.5 mmol), toluene (3 mL), 120 °C.

**Table 2:** Ruthenium-catalyzed *N*-alkylation of arylamines with arylmethyl alcohol ^1^.

Entry	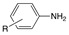	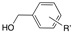	Conversion ^2^ (%)	Product Distribution ^2^ (Amine 14/Imine 13)
1	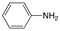	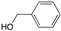	99	99/1
2	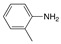	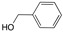	17	95/5
3		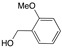	24	100/0
4	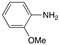	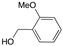	64	100/0
5	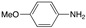	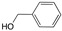	95	100/0
6		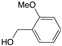	94	100/0

^1^ Reaction conditions: ruthenium complex **2** (1 mol%), arylmethyl alcohol (2 mmol), arylamine (1 mmol), tBuOK (1 mmol), 120 °C, 24 h. ^2^ The conversions and product distributions were determined by GC.

**Table 3:** Crystal data and structure refinement parameters for the ruthenium complex **3**.

CCDC depository		2409059	chemical formula	C_62_H_80_Cl_2_NO_8_Ru•(CHCl_3_)1.15
color/shape		orange/prism	formula weight (g mol^−1^)	1276.51
crystal system		triclinic	space group	*P*-1
unit cell parameters	*a* (Å)	11.1926(4)	volume (Å^3^)	3566.8(7)
	*b* (Å)	12.0728(4)	*Z*	2
	*c* (Å)	26.0769(7)	D (g cm^−3^)	1.357
	*α* (°)	77.7210(10)	μ (mm^−1^)	0.537
	*β* (°)	82.6390(10)	*T*_min_, *T*_max_	0.929/0.948
	*γ* (°)	65.2670(10)	*F*(000)	1335
crystal size (mm)		0.140 × 0.120 × 0.100	index ranges	−14 ≤ *h* ≤ 14
*θ* range for data collection (°)		2.005 ≤ *θ* ≤ 27.939		−15 ≤ *k* ≤ 15
reflections collected		129779		−34 ≤ *l* ≤ 32
data/restraints/parameters		14968/9/755	goodness-of-fit on *F*^2^	1.029
final R indices (*I* > 2.0 *σ*(*I*))		*R*_1_ = 0.0411	R indices (all data)	*R*_1_ = 0.0487
		*wR*_2_ = 0.0941		*wR*_2_ = 0.0996
Δ*ρ*_max_, Δ*ρ*_min_ (e Å^−3^)		1.989, −1.343		

## Data Availability

The data presented in this study are available upon request from the corresponding authors.

## Supplementary Materials

The following supporting information can be downloaded at: https://www.mdpi.com/article/10.3390/molecules30040951/s1. Characterizing data of 5,17-diazido-4(24),6(10),12(16),18(22)-tetramethyl-enedioxy-2,8,14,20-tetrapentyl-resorcin[4]arene (**9**), Figure S1. FT-IR spectrum, Figure S2. ^1^H NMR spectrum, Figure S3. ^13^C{^1^H} NMR spectrum; characterizing data of 5,11-diazido-4(24),6(10),12(16),18(22)-tetramethylenedioxy-2,8,14,20-tetrapentyl-resorcin[4]arene (**10**), Figure S4. FT-IR spectrum, Figure S5. ^1^H NMR spectrum, Figure S6. ^13^C{^1^H} NMR spectrum; characterizing data of 5,17-diamino-4(24),6(10),12(16),18(22)-tetramethylenedioxy-2,8,14,20-tetra-pentyl-resorcin[4]arene (**4**), Figure S7. FT-IR spectrum, Figure S8. ^1^H NMR spectrum, Figure S9. ^13^C{^1^H} NMR spectrum; characterizing data of 5,11-diamino-4(24),6(10),12(16),18(22)-tetramethyl-enedioxy-2,8,14,20-tetrapentyl-resorcin[4]arene (**5**), Figure S10. FT-IR spectrum, Figure S11. ^1^H NMR spectrum, Figure S12. ^13^C{^1^H} NMR spectrum; characterizing data of *N*,*N′*-{5,17-diamino-4(24),6(10),12(16),18(22)-tetramethylenedioxy-2,8,14,20-tetrapentylresorcin[4] arene}-bis-[dichloro-(*p*-cymene)-ruthenium(II)] (**1**), Figure S13. FT-IR spectrum, Figure S14. ^1^H NMR spectrum, Figure S15. ^13^C{^1^H} NMR spectrum, Figure S16. Mass spectrum (ESI-TOF) and X-Ray Crystal Structure Analysis of complex **1,** Table S1. Crystal data and structure refinement parameters for the ruthenium complex **1,** Figure S17. ORTEP drawing of ruthenium(II) complex **1**; characterizing data of N,N′-{5,11-diamino-4(24),6(10),12(16),18(22)-tetramethylenedioxy-2,8,14,20-tetrapentylresorcin[4]arene}-bis-[dichloro-(*p*-cymene)-ruthenium(II)] (**2**), Figure S18. FT-IR spectrum, Figure S19. ^1^H NMR spectrum, Figure S20. ^13^C{^1^H} NMR spectrum, Figure S21. Mass spectrum (ESI-TOF); characterizing data of *N*-{5-amino-4(24),6(10),12(16),18(22)-tetramethylenedioxy-2,8,14,20-tetrapentyl-resorcin[4]arene}-[dichloro-(*p*-cymene)-ruthenium(II)] (**3**), Figure S22. FT-IR spectrum, Figure S23. ^1^H NMR spectrum, Figure S24. ^13^C{^1^H} NMR spectrum, Figure S25. Mass spectrum (ESI-TOF), Figure S26. Mass spectrum (ESI-TOF); NMR description of the catalytic products.
