# Supplementary material for: Effect of the Positioning of Metal Centers on a Cavitand in the Ruthenium-Catalyzed N-Alkylation of Amines
Source: Molecules. 2025 Feb 18;30(4):951. doi: 10.3390/molecules30040951 (PMC11858491; doi:10.3390/molecules30040951)
Supplement: Supplementary file 1 [file molecules-30-00951-s001.zip › molecules-3452683-supplementary.pdf]

Effect of the positioning of metal centers on a cavitand in the ruthenium-catalyzed *N*-alkylation of amines

Neslihan Şahin, Christophe Gourlaouen and David Sémeril

Contents

Characterizing data of 5,17-diazido-4(24),6(10),12(16),18(22)-tetramethylenedioxy-2,8,14,20-tetrapentyl-resorcin[4]arene (**9**) p 2

Characterizing data of 5,11-diazido-4(24),6(10),12(16),18(22)-tetramethylenedioxy-2,8,14,20-tetrapentyl-resorcin[4]arene (**10**) p 4

Characterizing data of 5,17-diamino-4(24),6(10),12(16),18(22)-tetramethylenedioxy-2,8,14,20-tetrapentyl-resorcin[4]arene (**4**) p 6

Characterizing data of 5,11-diamino-4(24),6(10),12(16),18(22)-tetramethylenedioxy-2,8,14,20-tetrapentyl-resorcin[4]arene (**5**) p 8

Characterizing data of *N,N'*-{5,17-diamino-4(24),6(10),12(16),18(22)-tetramethylenedioxy-2,8,14,20-tetrapentylresorcin[4]arene}-bis-[dichloro-(*p*-cymene)-ruthenium(II)] (**1**) p 10

Characterizing data of *N,N'*-{5,11-diamino-4(24),6(10),12(16),18(22)-tetramethylenedioxy-2,8,14,20-tetrapentylresorcin[4]arene}-bis-[dichloro-(*p*-cymene)-ruthenium(II)] (**2**) p 14

Characterizing data of *N*-{5-amino-4(24),6(10),12(16),18(22)-tetramethylenedioxy-2,8,14,20-tetrapentyl-resorcin[4]arene}-[dichloro-(*p*-cymene)-ruthenium(II)] (**3**) p 17

NMR description of the catalytic products p 20

5,17-Diazido-4(24),6(10),12(16),18(22)-tetramethylenedioxy-2,8,14,20-tetrapentyl-resorcin[4]arene (9)

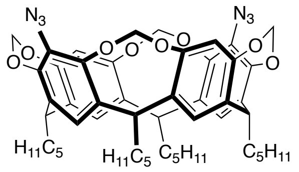

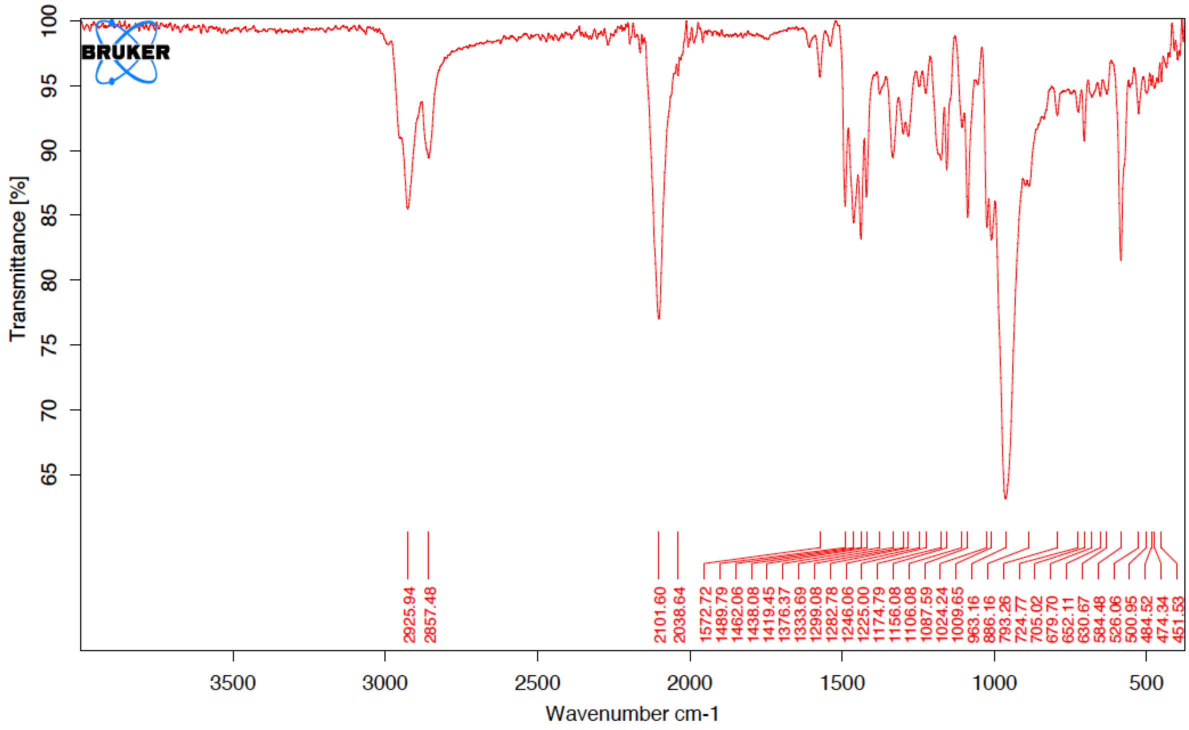

Figure S1. FT-IR spectrum

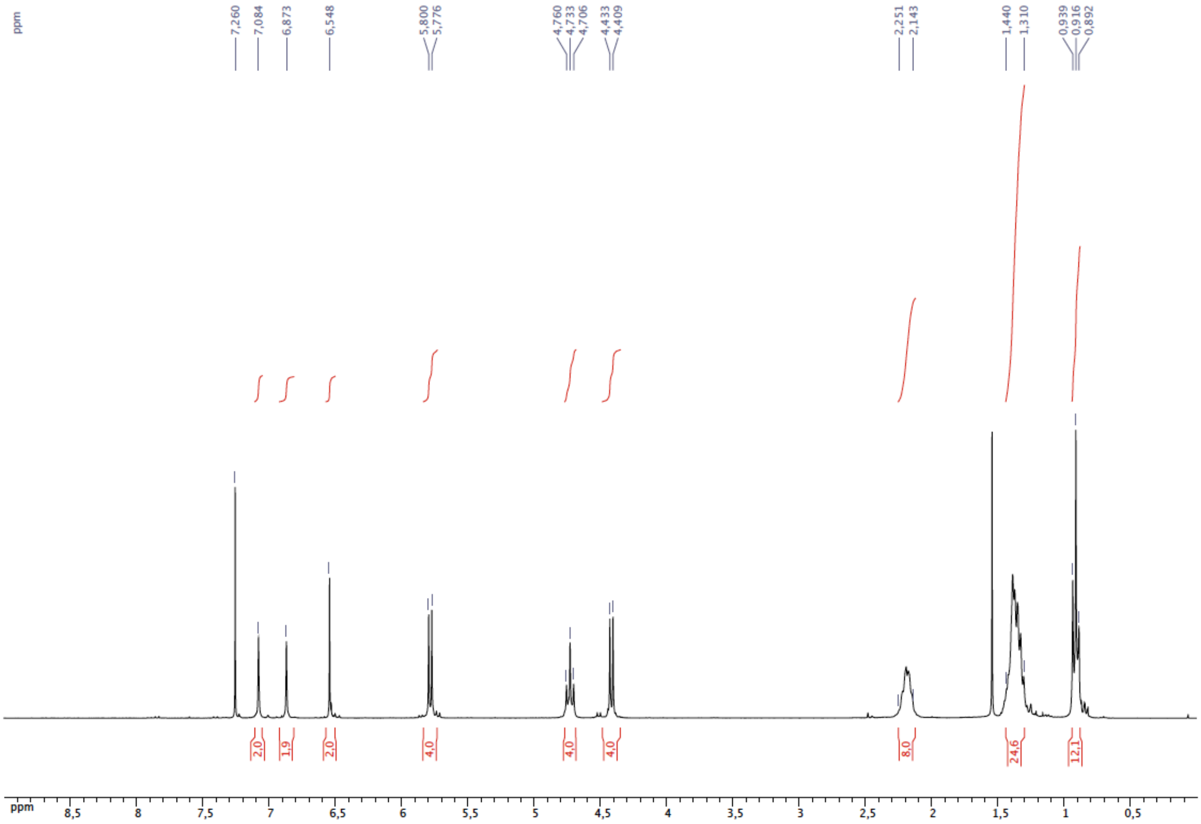

Figure S2. ^{1}H NMR spectrum ($CDCl_3$)

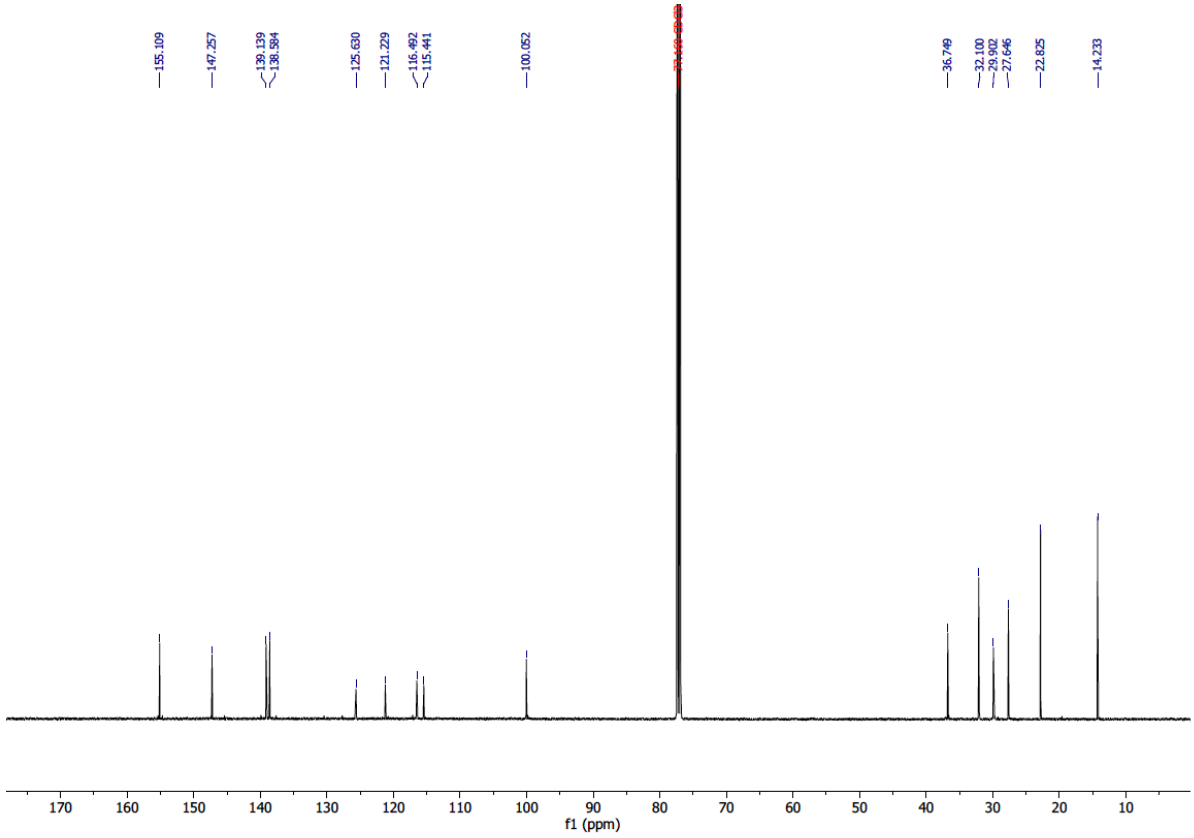

Figure S3. $^{13}C\{^{1}H\}$ NMR spectrum ($CDCl_3$)

5,11-Diazido-4(24),6(10),12(16),18(22)-tetramethylenedioxy-

2,8,14,20-tetrapentyl-resorcin[4]arene (10)

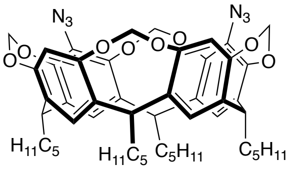

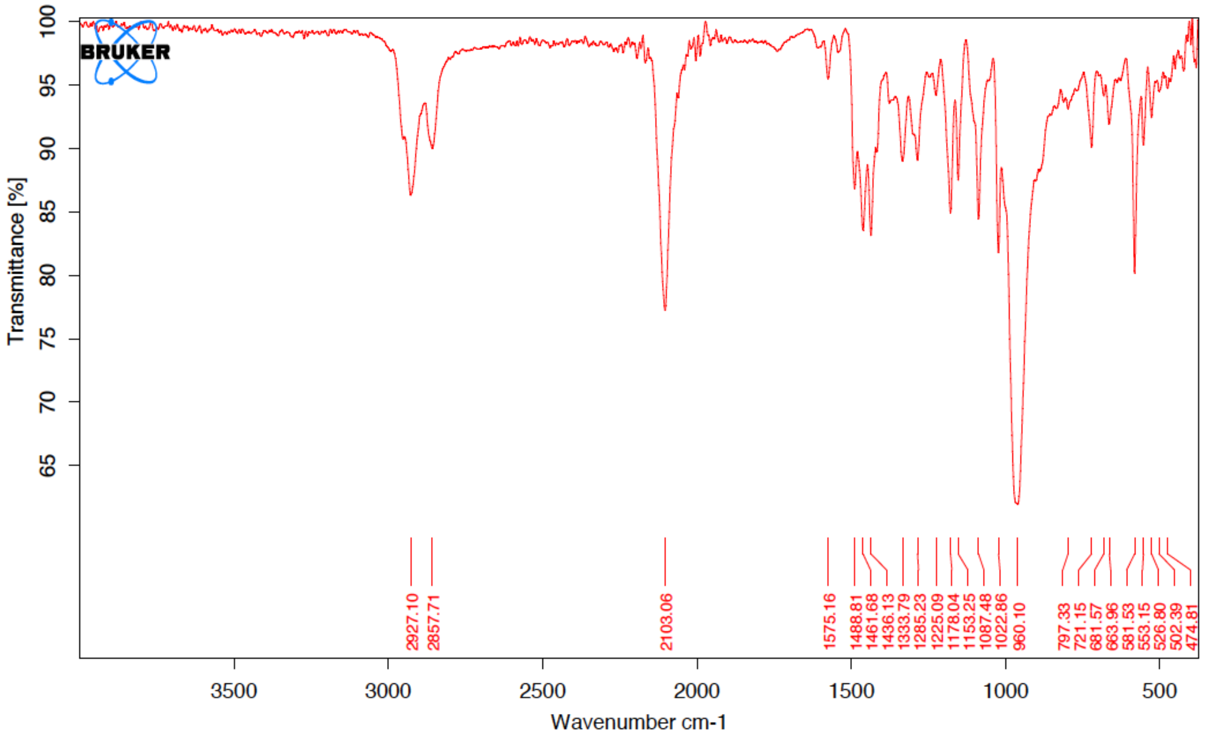

Figure S4. FT-IR spectrum

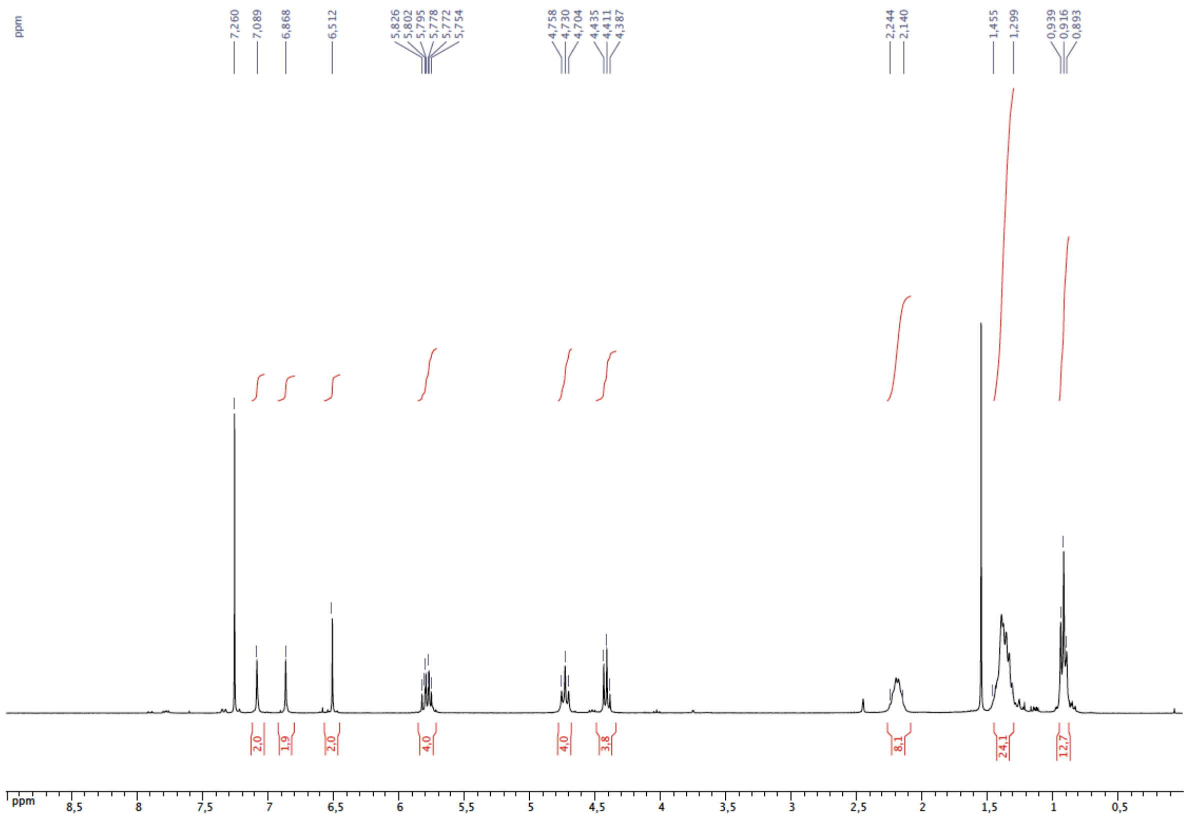

Figure S5. ^{1}H NMR spectrum ($CDCl_3$)

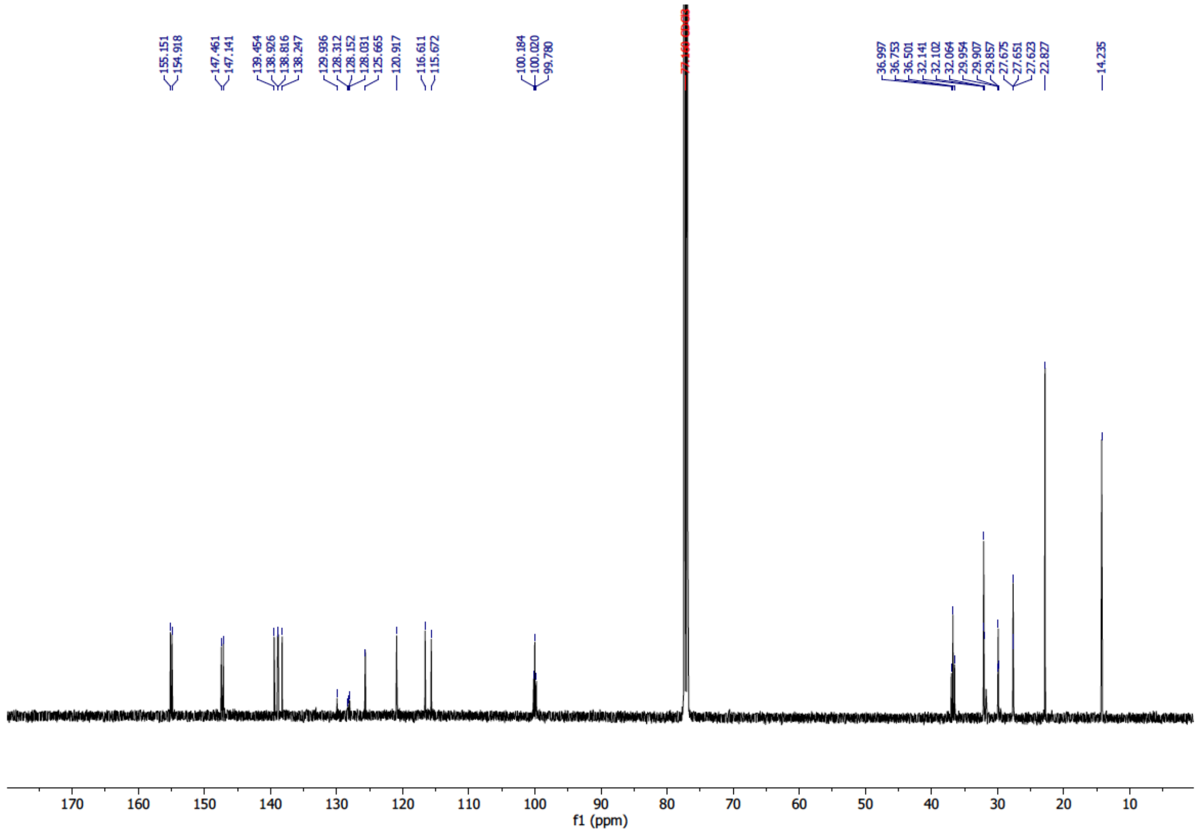

Figure S6. $^{13}C\{^{1}H\}$ NMR spectrum ($CDCl_3$)

5,17-Diamino-4(24),6(10),12(16),18(22)-tetramethylenedioxy-2,8,14,20-tetrapentyl-resorcin[4]arene (4)

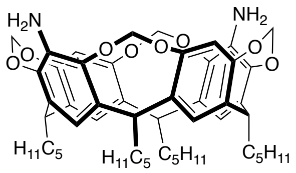

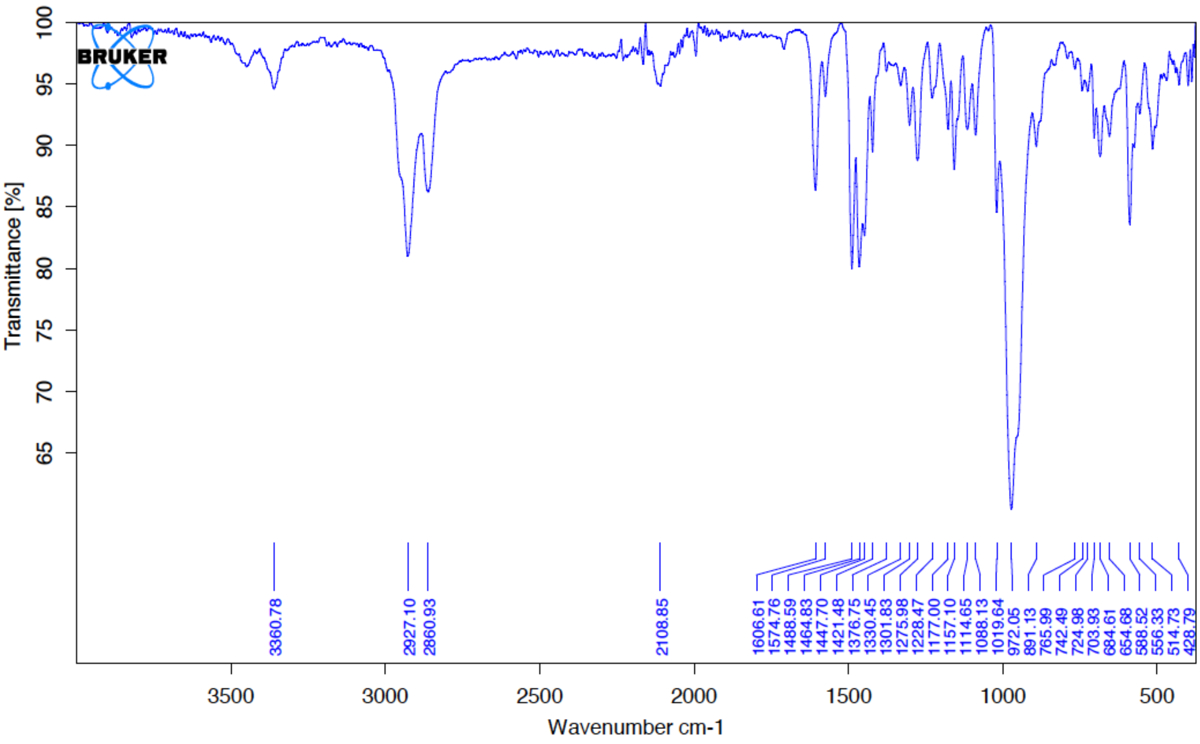

Figure S7. FT-IR spectrum

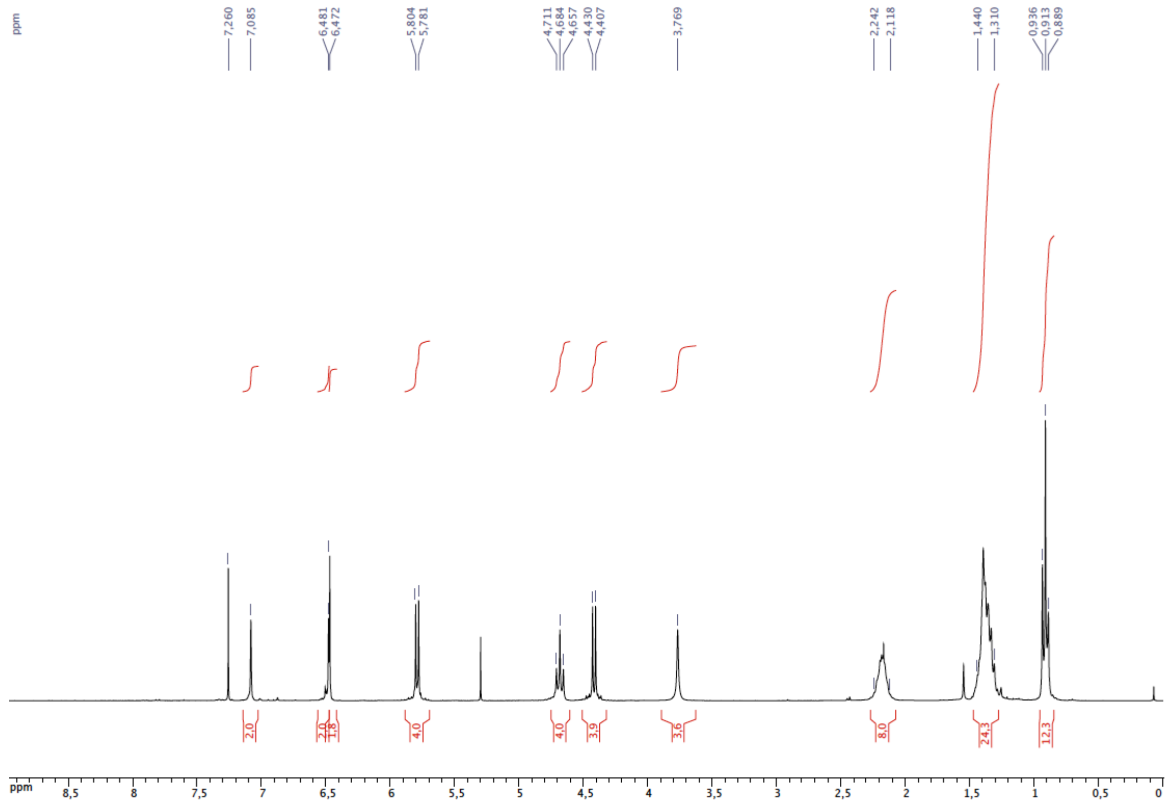

Figure S8. ^{1}H NMR spectrum ($CDCl_3$)

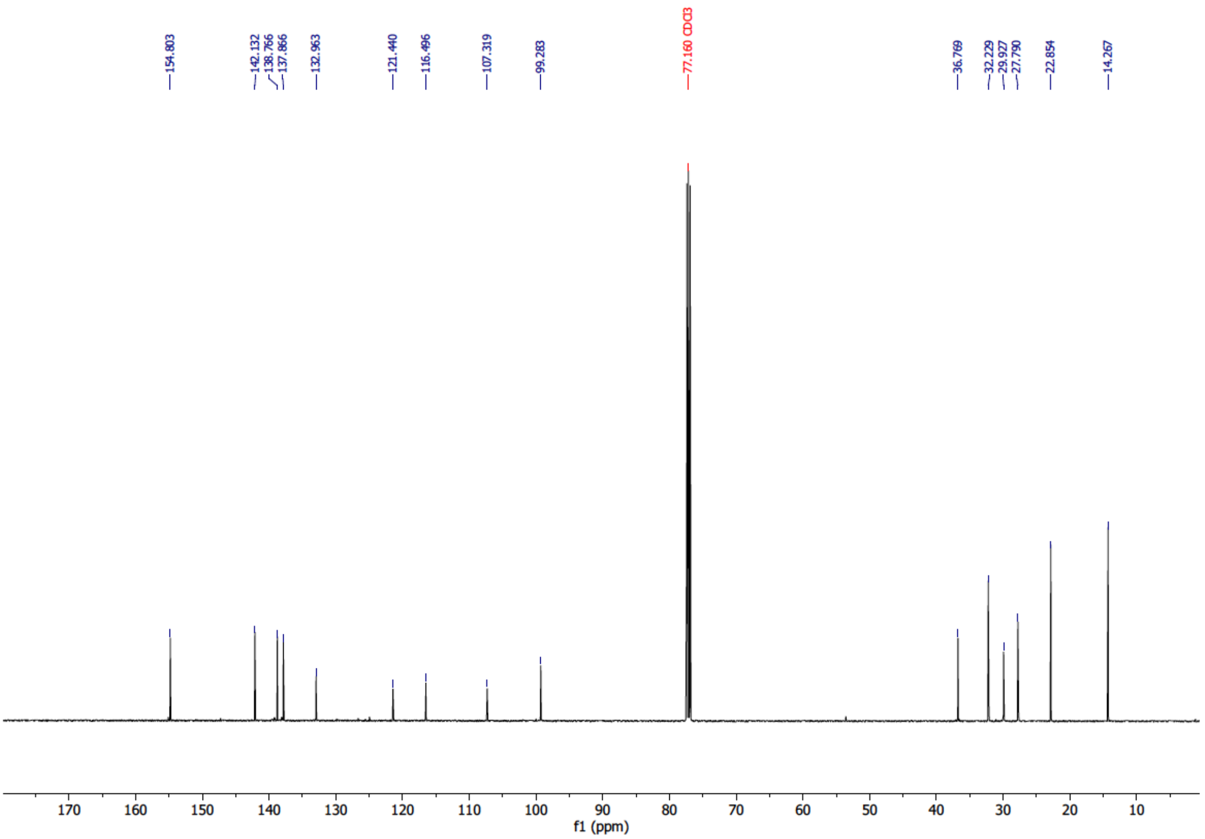

Figure S9. $^{13}C\{^{1}H\}$ NMR spectrum ($CDCl_3$)

5,11-Diamino-4(24),6(10),12(16),18(22)-tetramethylenedioxy-2,8,14,20-tetrapentyl-resorcin[4]arene (5)

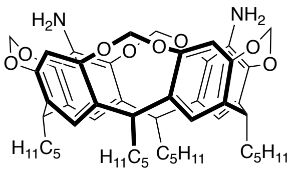

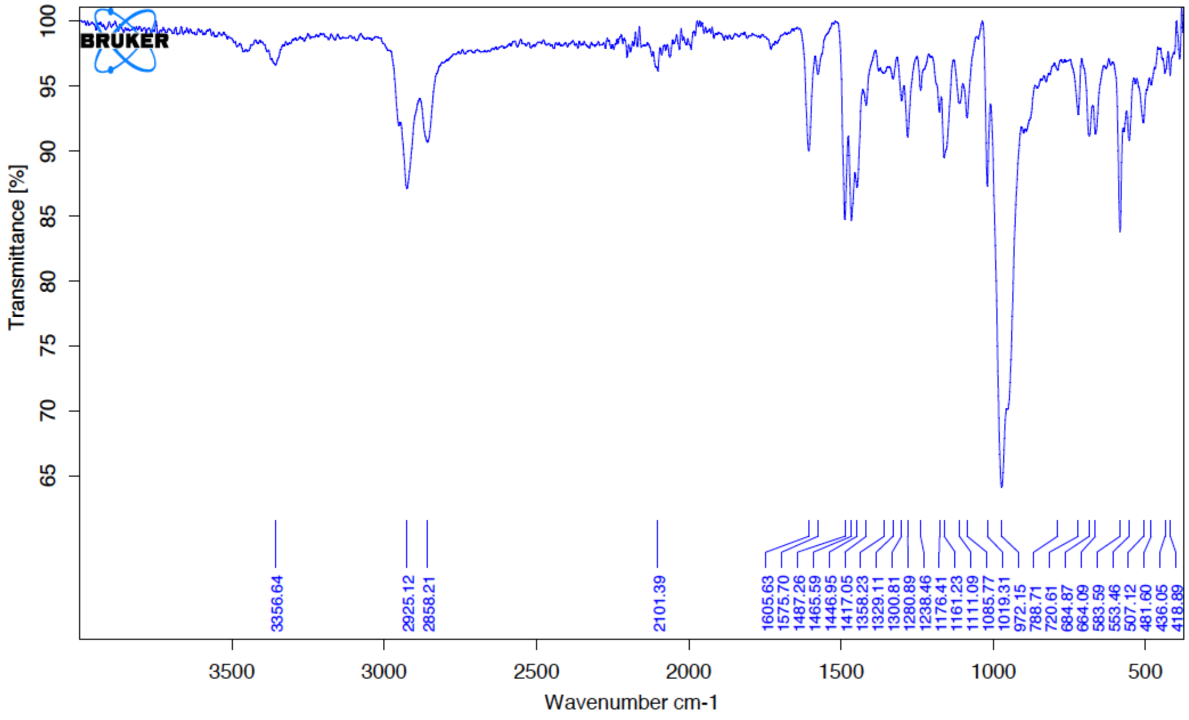

Figure S10. FT-IR spectrum

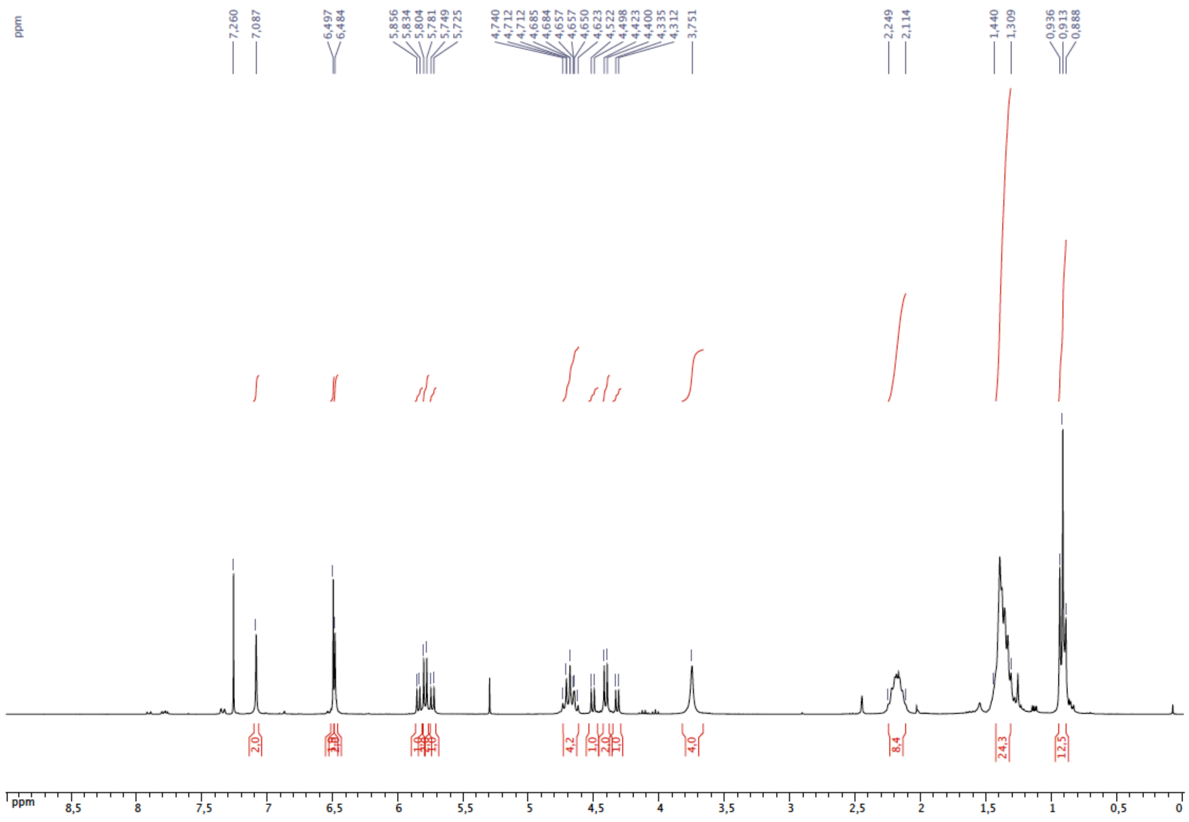

Figure S11. ^{1}H NMR spectrum ($CDCl_3$)

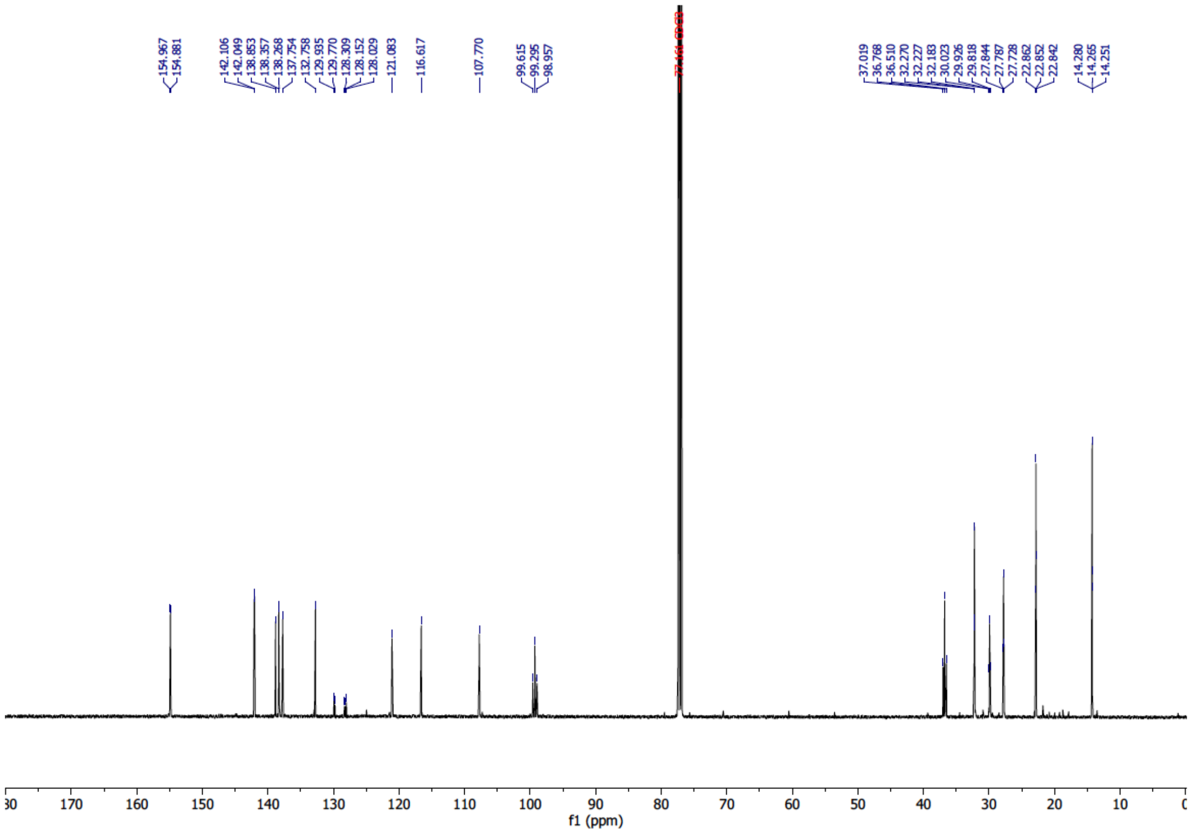

Figure S12. $^{13}C\{^{1}H\}$ NMR spectrum ($CDCl_3$)

***N,N'*-{5,17-Diamino-4(24),6(10),12(16),18(22)-tetramethylenedioxy-2,8,14,20-tetrapentylresorcin[4]arene}-bis-[dichloro-(*p*-cymene)-ruthenium(II)] (1)**

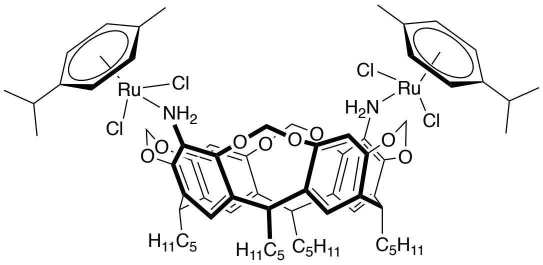

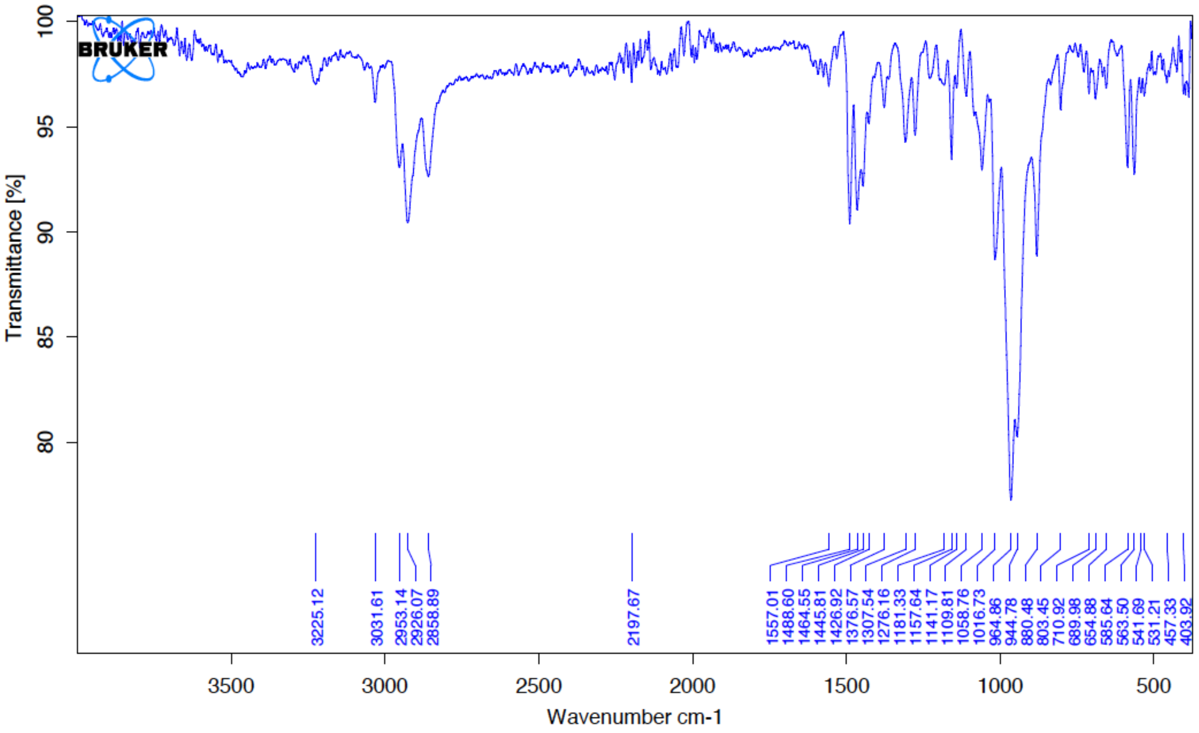

Figure S13. FT-IR spectrum

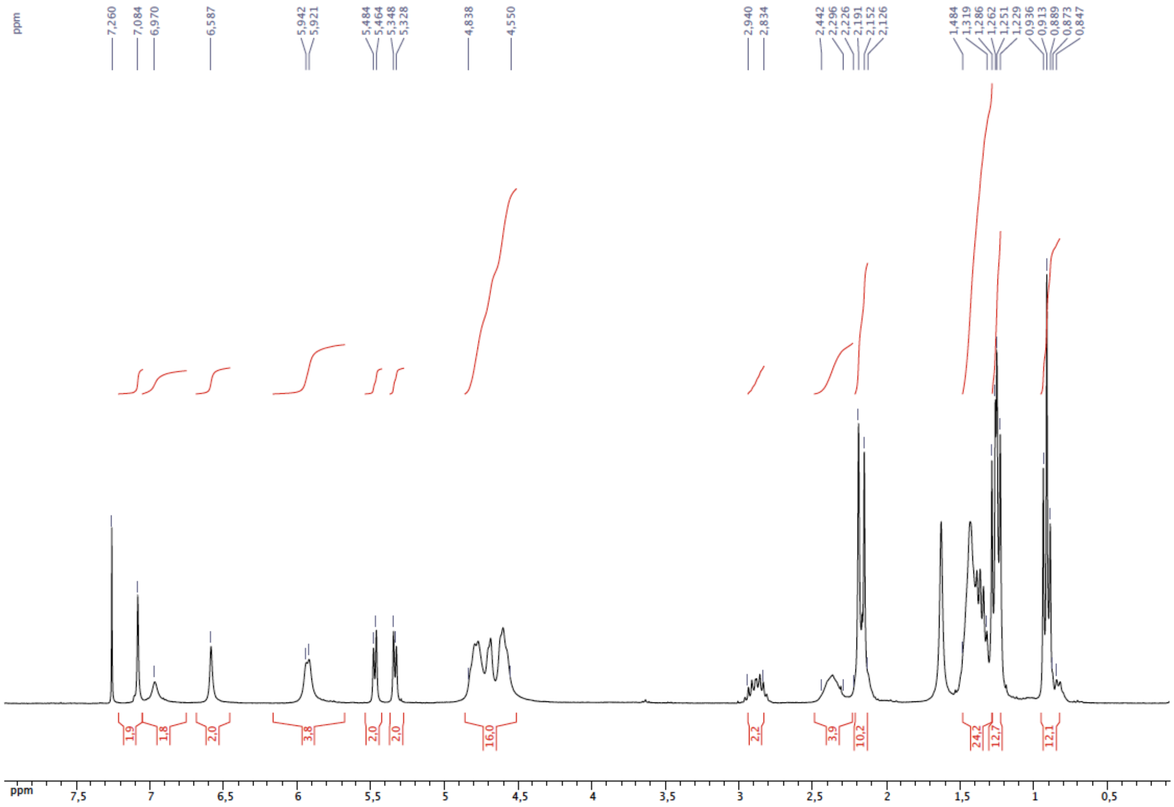

Figure S14. ^{1}H NMR spectrum ($CDCl_3$)

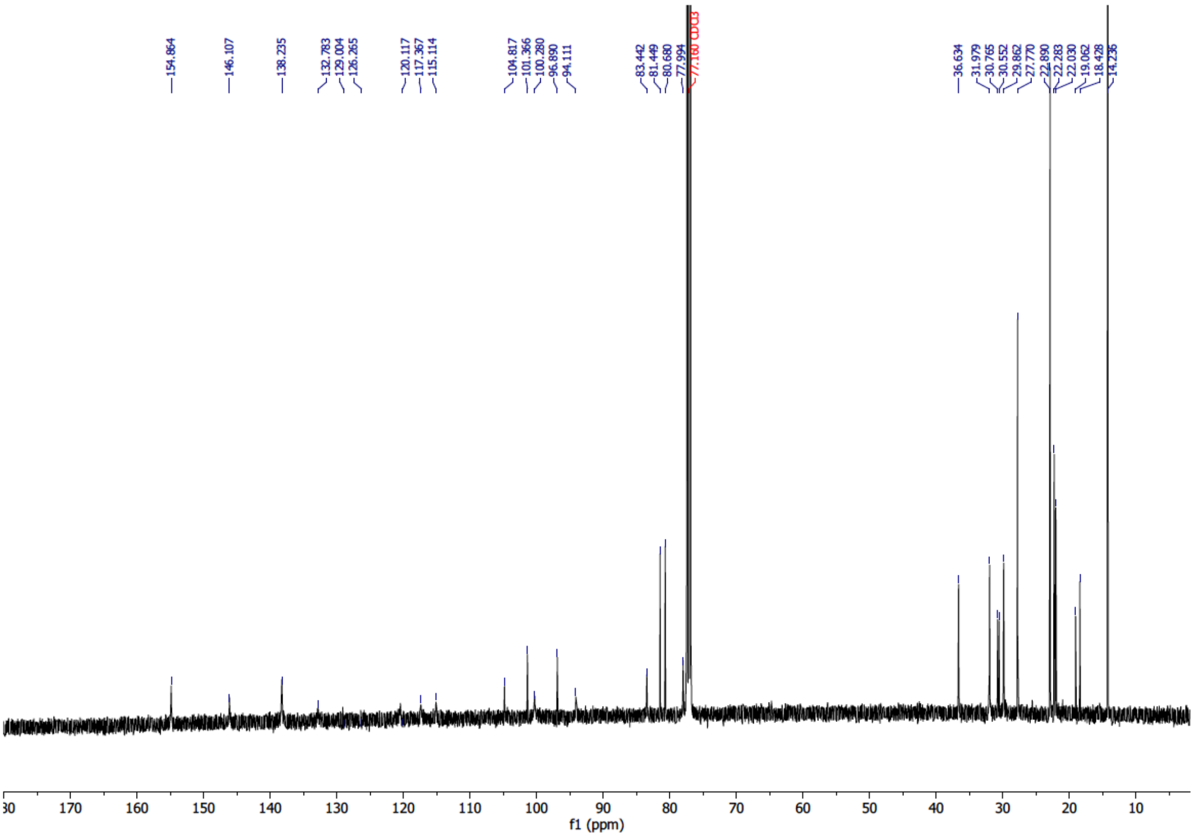

Figure S15. $^{13}C\{^{1}H\}$ NMR spectrum ($CDCl_3$)

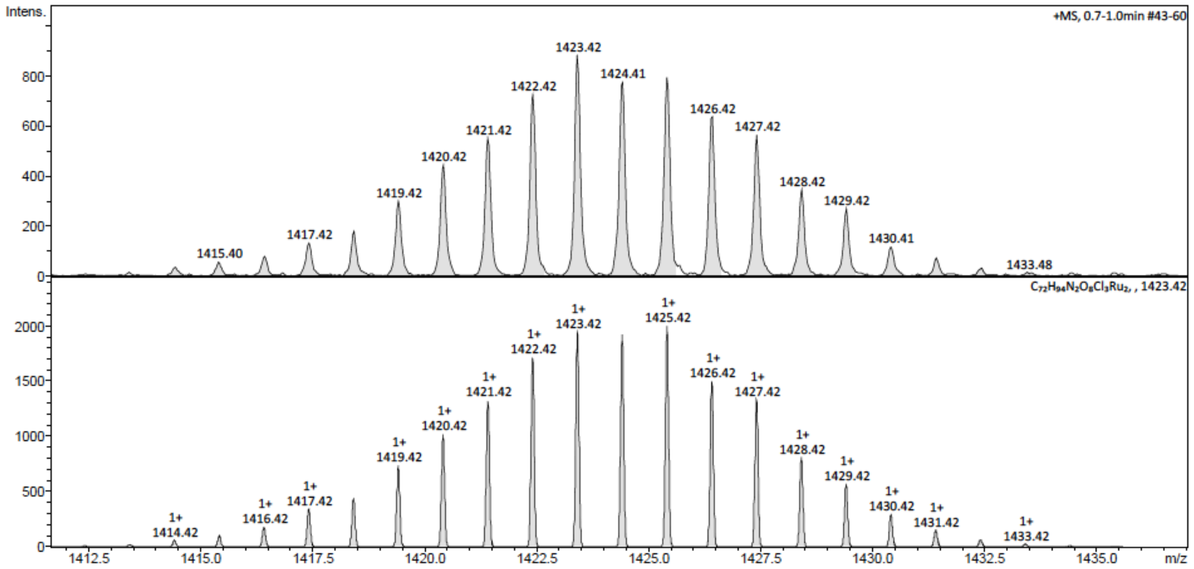

Figure S16. Mass spectrum (ESI-TOF)
exp. spectrum (top); calc. spectrum (bottom) for $C_{72}H_{94}O_8Ru_2Cl_3N_2$

X-Ray Crystal Structure Analysis of complex 1

Single crystals of ruthenium(II) complex **1**, suitable for X-ray analysis, were obtained by slow diffusion of Et_2O into a $CHCl_3$ solution of the complex. The samples were studied on a Bruker PHOTON-III CPAD, using Mo-Kα radiation (λ = 0.71073 Å) at T = 120(2) K. The structures were solved with SHELXT-2018/2,[1] which revealed the non-hydrogen atoms of the molecule. After anisotropic refinement, all of the hydrogen atoms were found with a Fourier difference map. The structure was refined with SHELXL-2019/2[2] by the full-matrix least-square techniques (use of F square magnitude; x, y, z, βij for C, Cl, N, O and Ru atoms; x, y, z in riding mode for H atoms). The four alkyl chains are disordered over two positions with for C33-C34 a ratio of 0.75/0.25, C37-C40 a ratio of 0.70/0.30, C45-C46 a ratio of 0.60/0.40, C50-C51 a ratio of 0.60/0.40, H32A and H32B a ratio of 0.75/0.25, H36A and H36B a ratio of 0.70/0.30 and H44A and H44B a ratio of 0.70/0.40. CCDC 2409060 contains the supplementary crystallographic data for this paper. The data can be obtained free of charge from The Cambridge Crystallographic Data Centre via www.ccdc.cam.ac.uk/structures.

[1] G. M. Sheldrick, *Acta Crystallogr. Sect. A* **2015**, *A71*, 3-8
[2] G. M. Sheldrick, *Acta Crystallogr. Sect. C* **2015**, *C71*, 3-8.

Table S1. Crystal data and structure refinement parameters for the ruthenium complex **1**.

CCDC depository		2409060	chemical formula	$C_{72}H_{94}Cl_4N_2O_8Ru_2$•$(CHCl_3)_2$
color /shape		orange / plate	formula weight (g mol^{-1})	1698.16
crystal system		triclinic	space group	*P*-1
unit cell parameters	*a* (Å)	12.3707(6)	volume (Å^3)	3566.8(7)
	b (Å)	18.6941(10)	Z	2
	c (Å)	20.7462(10)	*D* (g cm^{-3})	1.295
	α (°)	107.386(2)	*μ* (mm^{-1})	0.701
	β (°)	104.432(2)	T_{min}, T_{max}	0.873 / 0.933
	γ (°)	95.620(2)	*F*(000)	1752
crystal size (mm)		0.200 x 0.160 x 0.100	index ranges	$-17 \leq h \leq 17$
θ range for data collection (°)		$1.896 \leq \theta \leq 30.267$		$-26 \leq k \leq 26$
reflections collected		213083		$-29 \leq l \leq 29$
independent / observed		25847 / 18081	R_{int}	0.0687
data / restraints / parameters		25847 / 79 / 917	goodness-of-fit on F^2	1.019
final *R* indices ($I > 2.0\ \sigma(I)$)		$R_1 = 0.0723$ $wR_2 = 0.1942$	*R* indices (all data)	$R_1 = 0.1061$ $wR_2 = 0.2244$
$\Delta\rho_{max}$, $\Delta\rho_{min}$ (e Å^{-3})		2.324, -1.991		

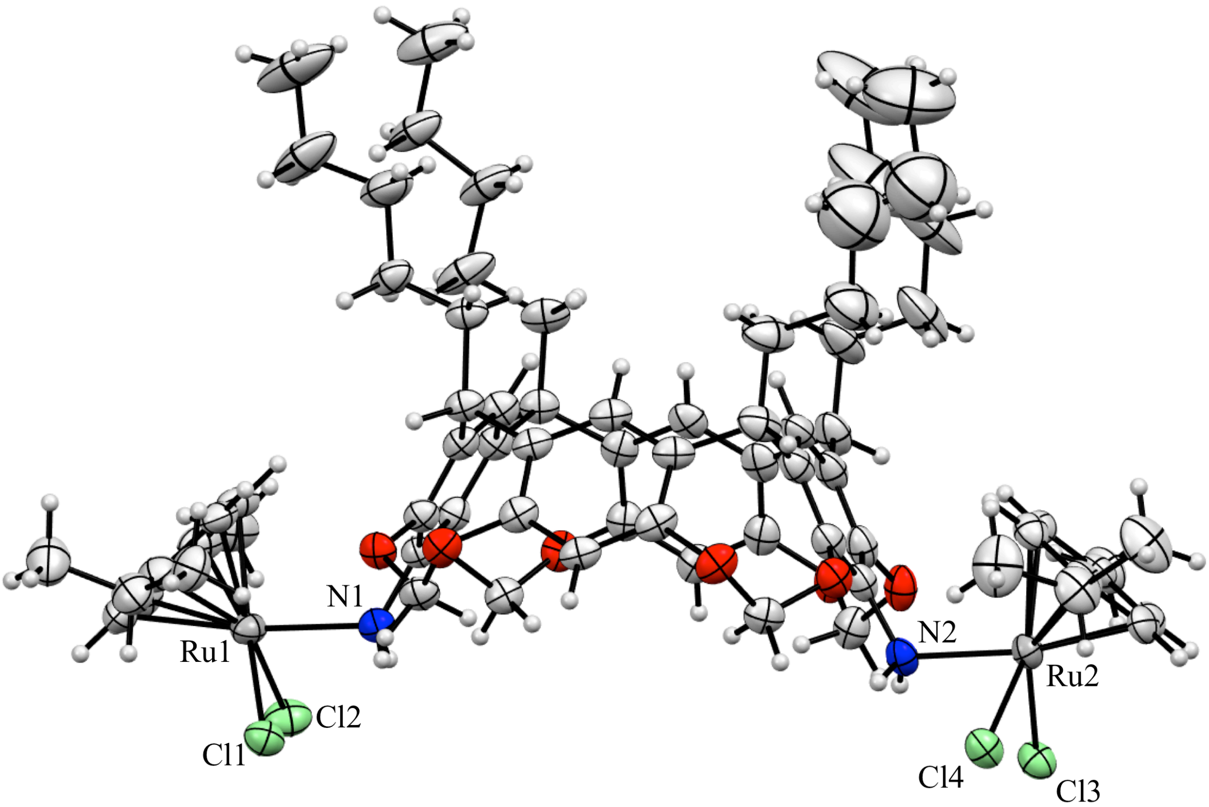

Figure S17. ORTEP drawing of ruthenium(II) complex **1**, 50 % probability thermal ellipsoids. For clarity, the 2 molecules of chloroform are not represented. Important bond lengths (Å) and angles (°): N1-Ru1 2.175(4), Ru1-Cl1 2.4202(12), Ru1-Cl2 2.4098(12), Ru1-C54 2.194(5), Ru1-C55 2.152(4), Ru1-C56 2.163(4), Ru1-C57 2.183(5), Ru1-C58 2.161(5), Ru1-C59 2.177(5), N2-Ru2 2.181(4), Ru2-Cl3 2.4090(11), Ru2-Cl4 2.3972(11), Ru2-C64 2.193(4), Ru2-C65 2.163(4), Ru2-C66 2.181(5), Ru2-C67 2.199(4), Ru2-C68 2.177(4), Ru2-C69 2.169(4), N1-Ru1-Cl1 79.77(11), Cl1-Ru1-Cl2 88.51(4), Cl2-Ru1-N1 81.33(11), N2-Ru2-Cl3 81.79(10), Cl3-Ru2-Cl4 86.66(4) and Cl4-Ru2-N2 81.99(12).

N,N'-{5,11-Diamino-4(24),6(10),12(16),18(22)-tetramethylenedioxy-2,8,14,20-tetrapentylresorcin[4]arene}-bis-[dichloro-(*p*-cymene)-ruthenium(II)] (2)

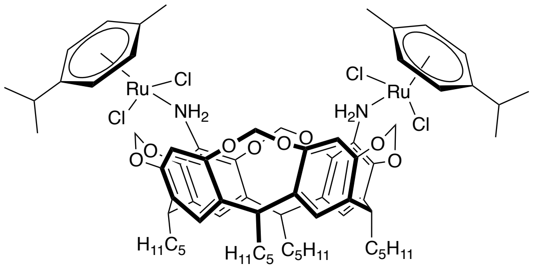

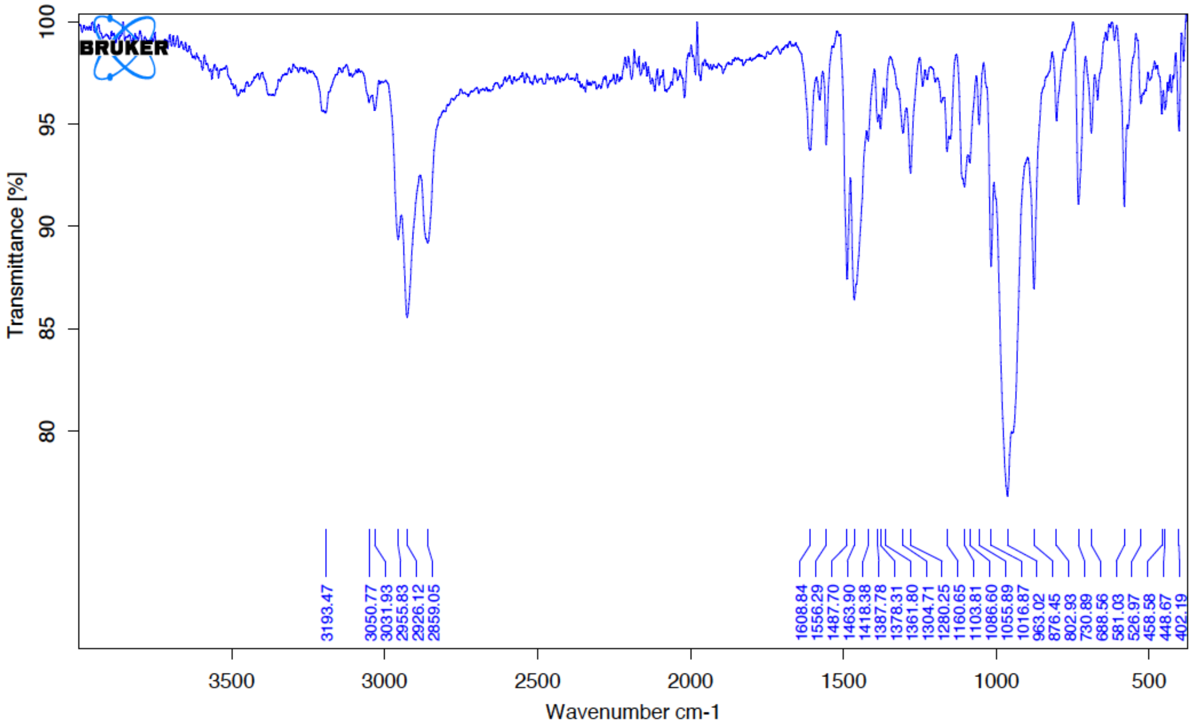

Figure S18. FT-IR spectrum

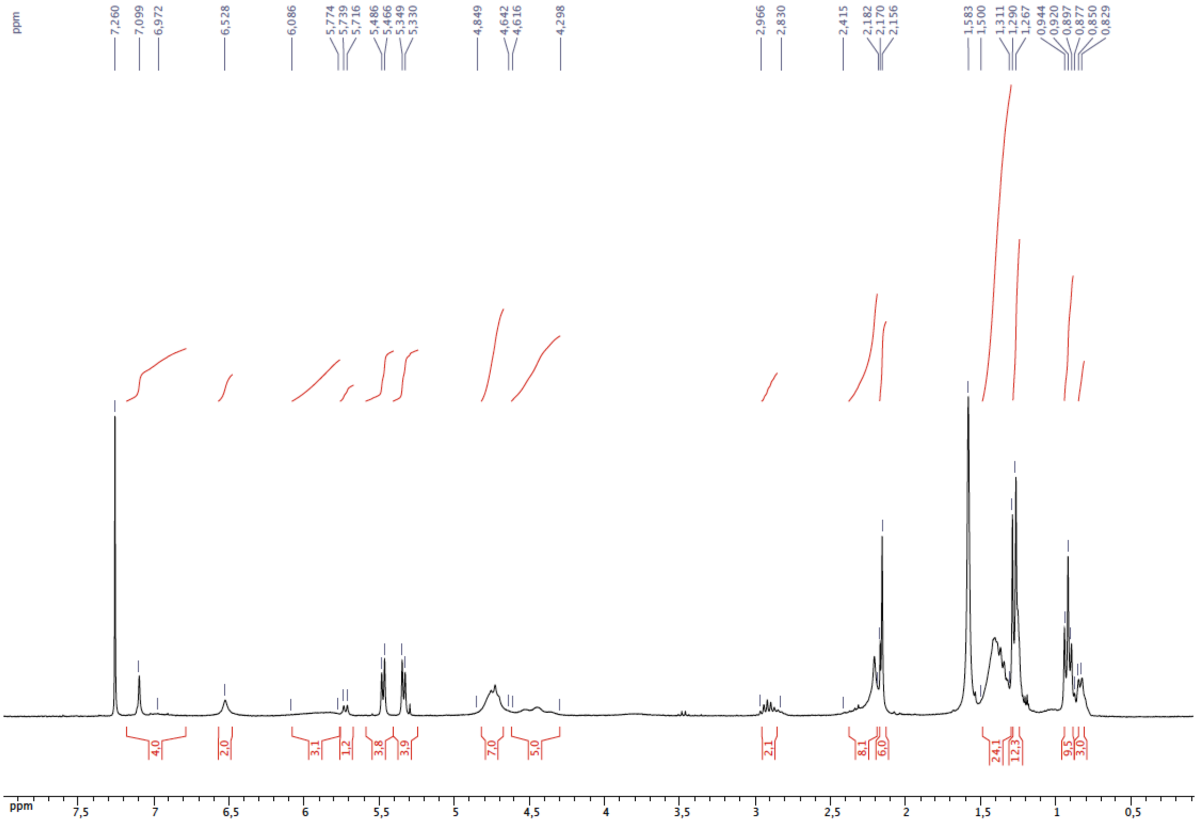

Figure S19. ^{1}H NMR spectrum ($CDCl_3$)

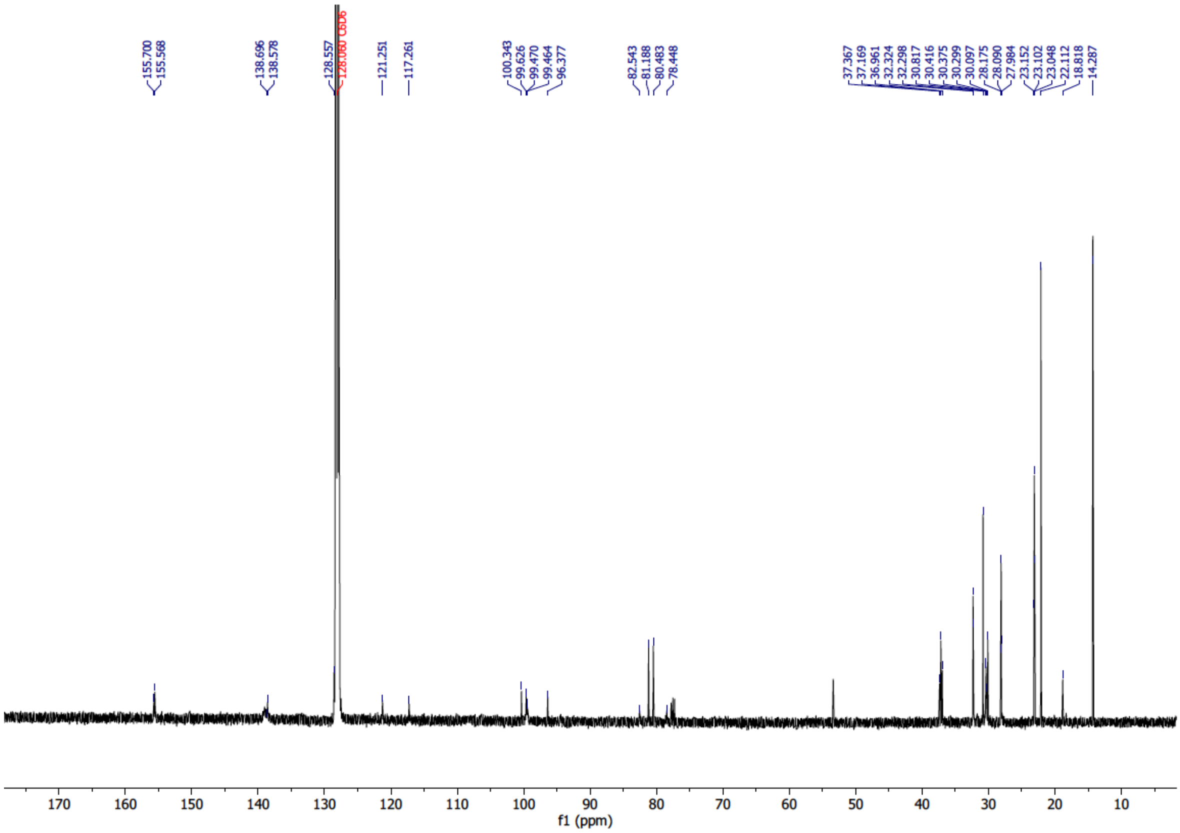

Figure S20. $^{13}C\{^{1}H\}$ NMR spectrum (C_6D_6)

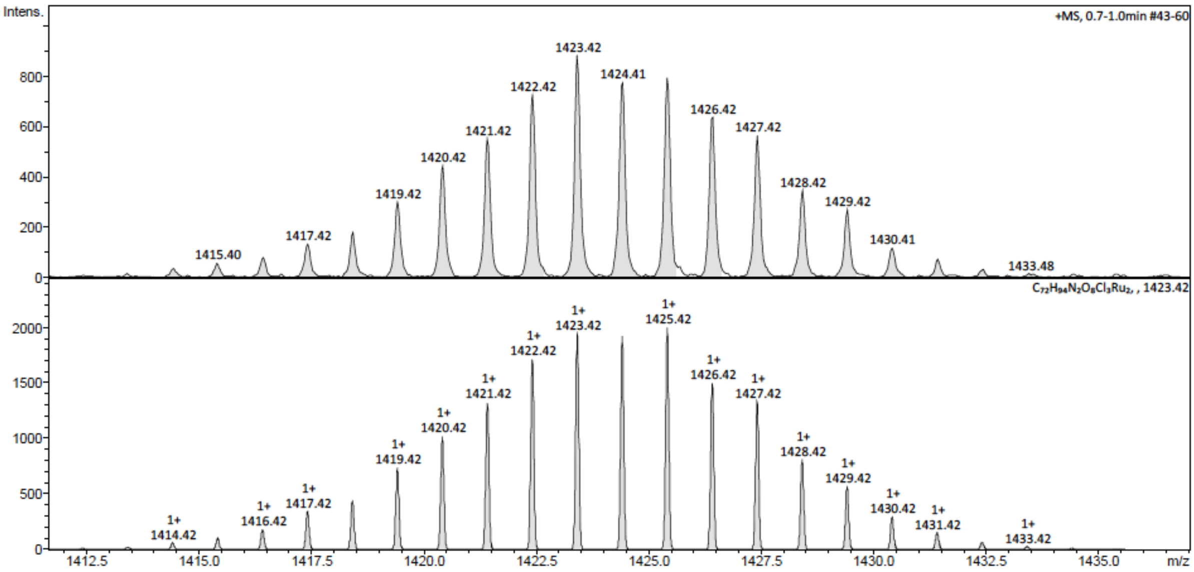

Figure S21. Mass spectrum (ESI-TOF)
exp. spectrum (top); calc. spectrum (bottom) for $C_{72}H_{94}O_8Ru_2Cl_3N_2$

_N_-{5-Amino-4(24),6(10),12(16),18(22)-tetramethylenedioxy-2,8,14,20-tetrapentyl-resorcin[4]arene}-[dichloro-(_p_-cymene)-ruthenium(II)] (3)

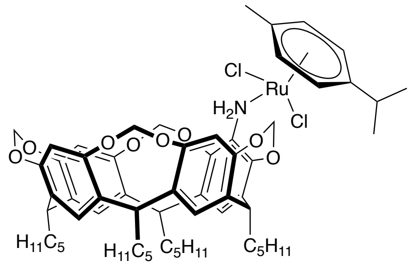

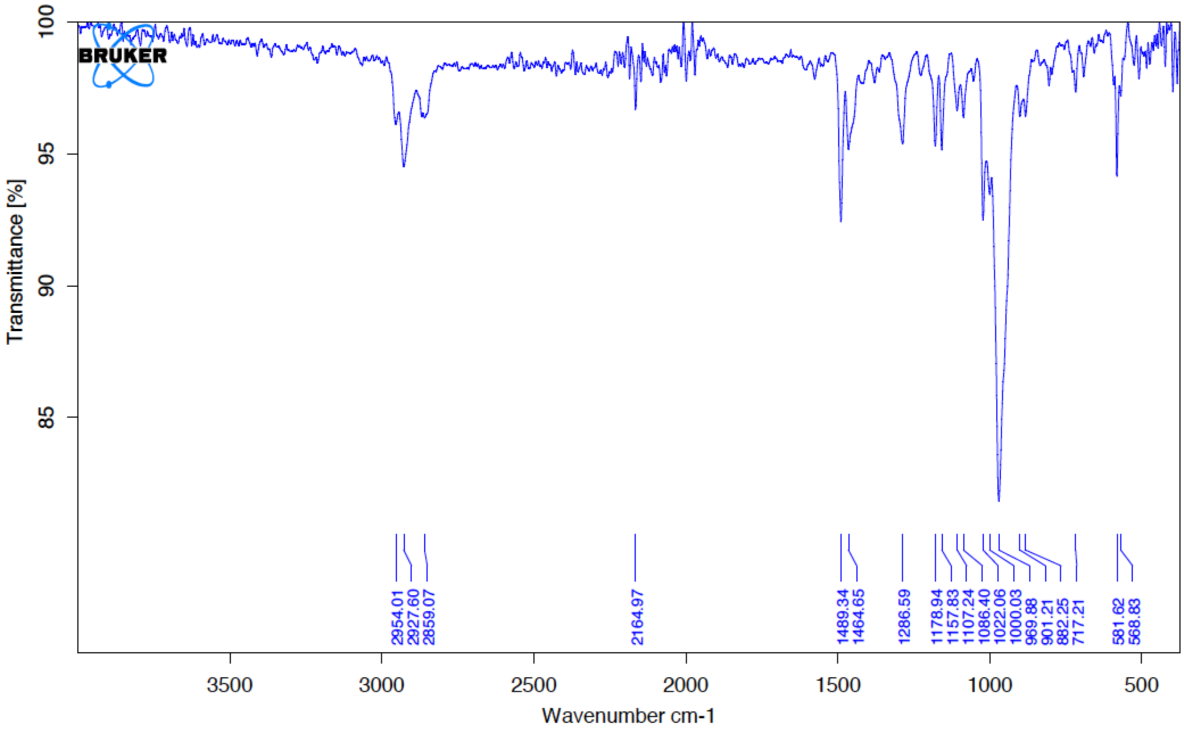

Figure S22. FT-IR spectrum

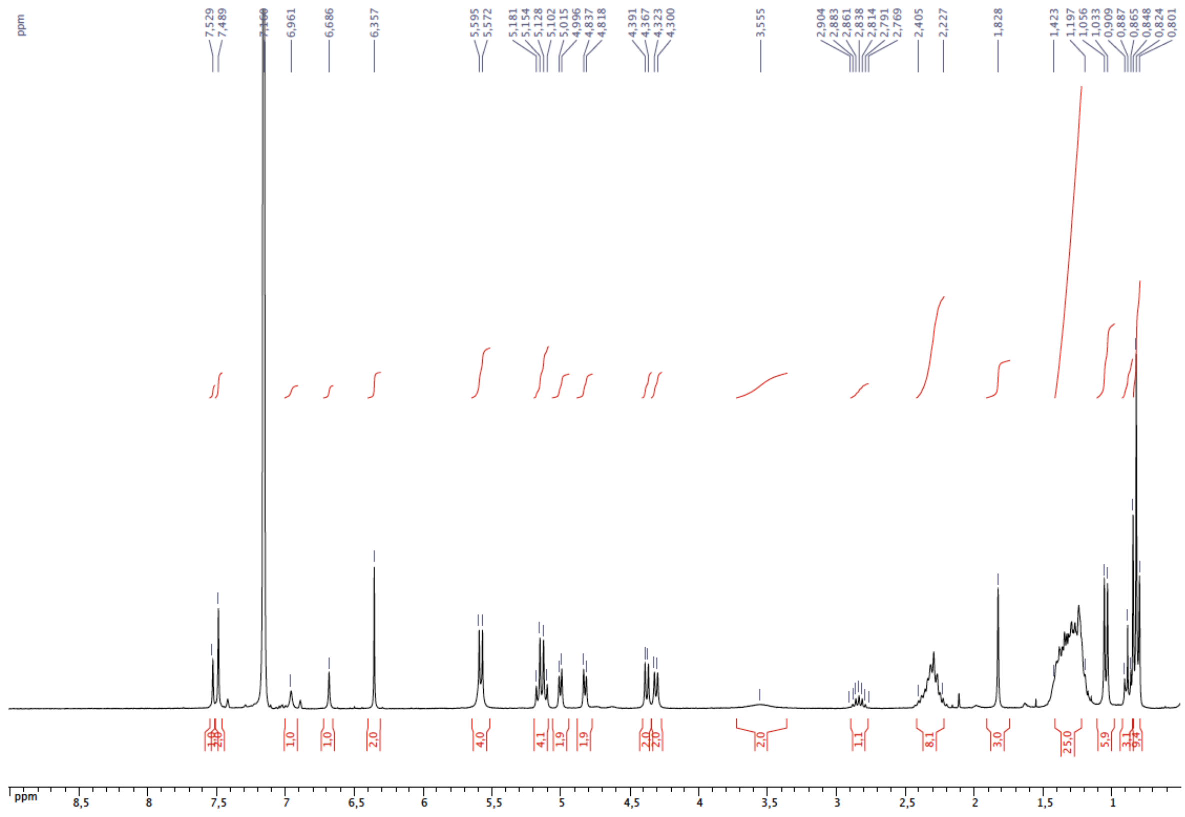

Figure S23. ^{1}H NMR spectrum (C_6D_6)

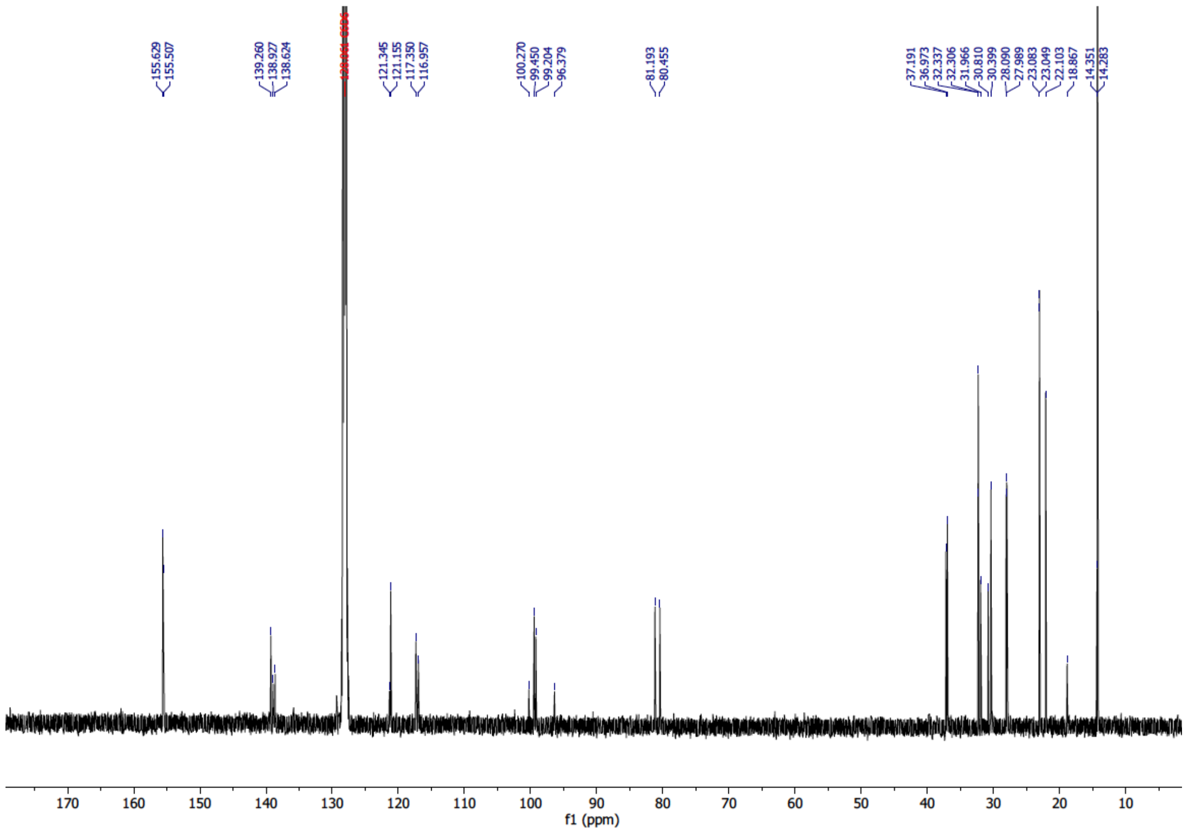

Figure S24. $^{13}C\{^{1}H\}$ NMR spectrum (C_6D_6)

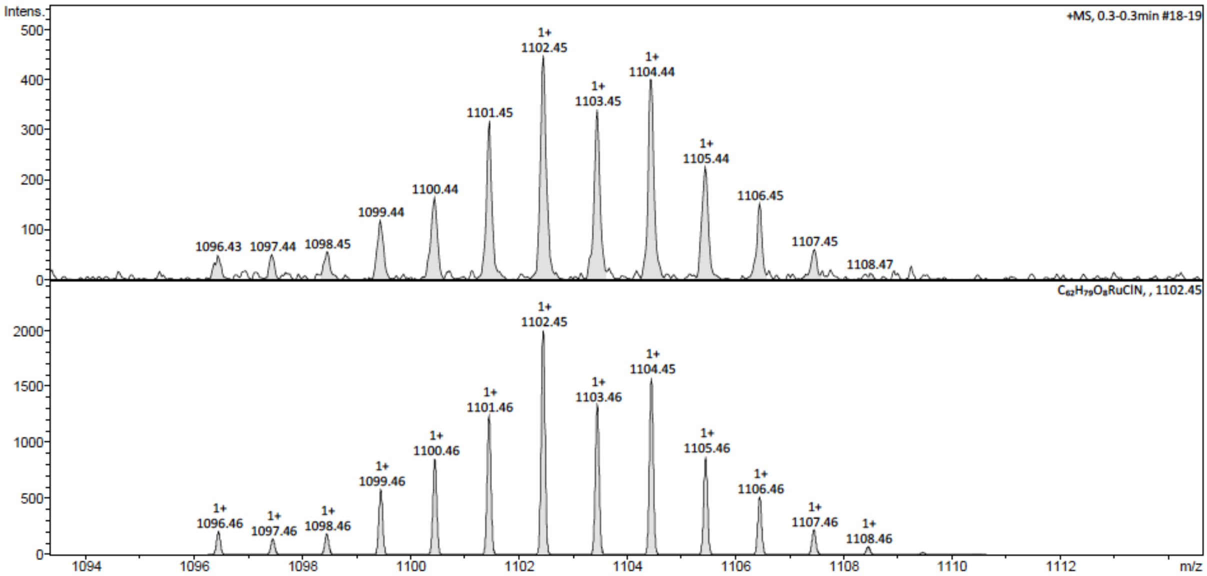

Figure S25. Mass spectrum (ESI-TOF)
exp. spectrum (top); calc. spectrum (bottom) for $C_{62}H_{79}O_8RuClN$

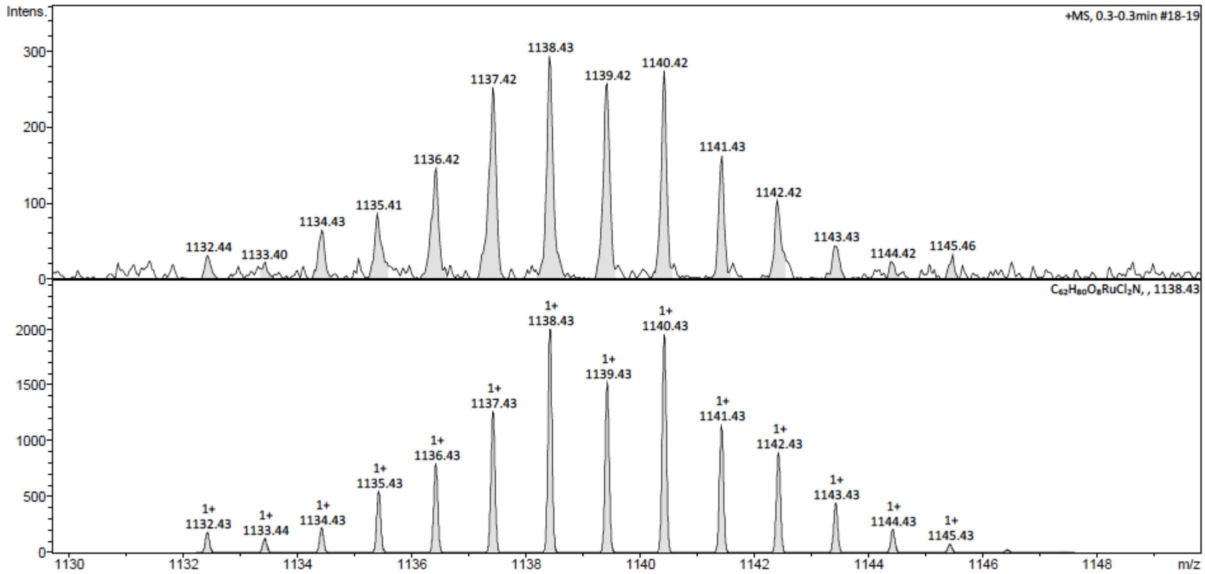

Figure S26. Mass spectrum (ESI-TOF)
exp. spectrum (top); calc. spectrum (bottom) for $C_{62}H_{80}O_8RuCl_2N$

^{1}H NMR description of the catalytic products

***N*-Benzylaniline (14a):** ^{1}H NMR (300 MHz, $CDCl_3$) δ = 7.40-7.31 (m, 4H, arom CH), 7.30-7.26 (m, 1H, arom CH), 7.16 (t, 2H, arom CH, $^{3}J_{HH}$ = 7.5 Hz), 6.70 (t, 1H, arom CH, $^{3}J_{HH}$ = 7.5 Hz), 6.64 (d, 2H, arom CH, $^{3}J_{HH}$ = 7.5 Hz), 4.31 (s, 2H, NCH_2); ^{13}C NMR (126 MHz, $CDCl_3$) δ = 148.20, 139.52, 129.38, 128.73, 127.57, 127.30, 117.66, 112.96 (8 s, arom. Cs), 48.41 (s, NCH_2) ppm.

***N*-Benzyl-2-methylaniline (14b):** ^{1}H NMR (300 MHz, $CDCl_3$) δ = 7.41-7.31 (m, 4H, arom CH), 7.30-7.26 (m, 1H, arom CH), 7.10-7.04 (m, 2H, arom CH), 6.71-6.64 (m, 1H, arom CH), 6.61 (d, 1H, arom CH, $^{3}J_{HH}$ = 7.7 Hz), 4.33 (s, 2H, NCH_2), 2.14 (s, 3H, CH_3); ^{13}C NMR (126 MHz, $CDCl_3$) δ = 146.21, 139.59, 130.17, 128.75, 127.66, 127.34, 127.29, 121.99, 117.30, 110.12 (10 s, arom. Cs), 48.41 (s, NCH_2), 17.66 (s, CH_3) ppm.

***N*-(2-Methoxybenzyl)-2-methylaniline (14c):** ^{1}H NMR (300 MHz, $CDCl_3$) δ = 7.40-7.29 (m, 2H, arom CH), 7.22-7.07 (m, 2H, arom CH), 7.03-6.95 (m, 2H, arom CH), 6.73-6.68 (m, 2H, arom CH), 4.44 (s, 2H, NCH_2), 3.95 (s, 3H, OCH_3), 2.21 (s, 3H, CH_3); ^{13}C NMR (126 MHz, $CDCl_3$) δ = 157.52, 146.44, 130.07, 128.93, 128.35, 127.48, 127.08, 122.12, 120.57, 116.95, 110.28, 110.25 (12 s, arom. Cs), 55.34 (s, OCH_3), 43.57 (s, NCH_2), 17.59 (s, CH_3) ppm.

***N*-(2-Methoxybenzyl)-2-methoxylaniline (14d):** ^{1}H NMR (300 MHz, $CDCl_3$) δ = 7.42-7.27 (m, 2H, arom CH), 7.08-7.90 (m, 3H, arom CH), 7.88-6.56 (m, 3H, arom CH), 4.46 (s, 2H, NCH_2), 3.93 (s, 3H, OCH_3), 3.80 (s, 3H, OCH_3); ^{13}C NMR (126 MHz, $CDCl_3$) δ = 157.37, 147.05, 138.39, 128.77, 128.16, 127.55, 121.41, 120.56, 116.35, 110.27, 110.12, 109.54 (12 s, arom. Cs), 55.47 (s, OCH_3), 55.38 (s, OCH_3), 42.87 (s, NCH_2) ppm.

***N*-Benzyl-4-methoxylaniline (14e):** ^{1}H NMR (300 MHz, $CDCl_3$) δ = 7.53-7.37 (m, 5H, arom CH), 6.93 (d, 2H, arom CH, $^{3}J_{HH}$ = 9.0 Hz), 6.68 (d, 2H, arom CH, $^{3}J_{HH}$ = 9.0 Hz), 4.37 (s, 2H, NCH_2), 3.90 (s, 3H, OCH_3); ^{13}C NMR (126 MHz, $CDCl_3$) δ = 152.17, 142.54, 139.81, 128.70, 127.66, 127.32, 115.04, 114.19 (8 s, arom. Cs), 49.48 (s, OCH_3), 44.87 (s, NCH_2) ppm.

***N*-(2-Methoxybenzyl)-4-methoxylaniline (14f):** ^{1}H NMR (300 MHz, $CDCl_3$) δ = 7.38-7.25 (m, 2H, arom CH), 7.01-6.89 (m, 2H, arom CH), 6.84 (d, 2H, arom CH, $^{3}J_{HH}$ = 9.0 Hz), 6.67 (d, 2H, arom CH, $^{3}J_{HH}$ = 9.0 Hz), 4.37 (s, 2H, NCH_2), 3.90 (s, 3H, OCH_3), 3.73 (s, 3H, OCH_3); ^{13}C NMR (126 MHz, $CDCl_3$) δ = 157.38, 152.07, 142.76, 128.91, 128.18, 127.56, 120.47, 114.83, 14.35, 110.24 (10 s, arom. Cs), 55.72 (s, OCH_3), 55.36 (s, OCH_3), 43.97 (s, NCH_2) ppm.
